# Site-Selective Functionalized PD-1 Mutant for a Modular Immunological Activity against Cancer Cells

**DOI:** 10.1021/acs.biomac.3c00893

**Published:** 2023-10-30

**Authors:** Silvia Fallarini, Linda Cerofolini, Maria Salobehaj, Domenico Rizzo, Giulia Roxana Gheorghita, Giulia Licciardi, Daniela Eloisa Capialbi, Valerio Zullo, Andrea Sodini, Cristina Nativi, Marco Fragai

**Affiliations:** † Department of Pharmaceutical Sciences, DSF, University of Piemonte Orientale, Largo Donegani 2, Novara (NO) 28100, Italy; ‡ Department of Chemistry, DICUS, University of Florence, Via della Lastruccia 3,13, Sesto Fiorentino (FI) 50019, Italy; § CeRM/CIRMMP, University of Florence, Via L. Sacconi 6, Sesto Fiorentino (FI) 50019, Italy; ∥ Giotto Biotech, S.R.L, Via Madonna del Piano 6, Sesto Fiorentino (FI) 50019, Italy

## Abstract

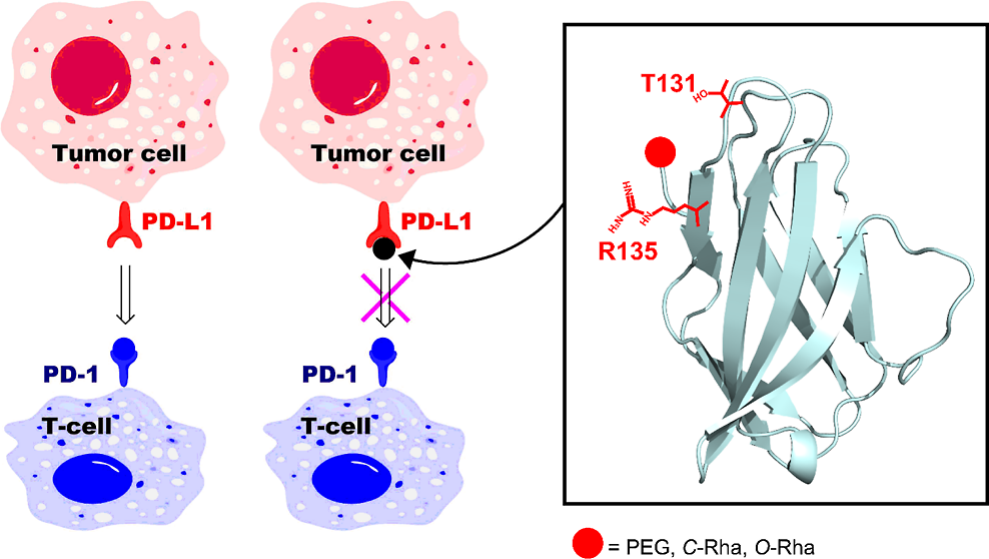

Targeting immune checkpoints is a well-established strategy in cancer therapy, and antibodies blocking PD-1/PD-L1 interactions to restore the immunological activity against cancer cells have been clinically validated. High-affinity mutants of the PD-1 ectodomain have recently been proposed as an alternative to antibodies to target PD-L1 on cancer cells, shedding new light on this research area. In this dynamic scenario, the PD-1 mutant, here reported, largely expands the chemical space of nonantibody and nonsmall-molecule inhibitor therapeutics that can be used to target cancer cells overexpressing PD-L1 receptors. The polyethylene glycol moieties and the immune response-stimulating carbohydrates, used as site-selective tags, represent the proof of concept for future applications.

## Introduction

T-cells are crucial components of the immune system involved in the adaptive response against pathogens and unhealthy cells, including cancer cells.^1−5^ Unfortunately, immune selection pressure often enables abnormal cells to deceive the immune system and escape immune surveillance. For example, many immunogenic tumors can bypass the immune destruction by exploiting checkpoints that are naturally deputed to regulate the immune system and suppress autoimmunity.^6^

One of these checkpoints modulates T-cell function through the activation of programmed cell death protein 1 (PD-1) and its physiological programmed cell death ligands, PD-L1 and PD-L2.^7,8^ Cancer cells overexpressing PD-L1 transmembrane protein neutralize the cytotoxic activity of T-cells, becoming free to replicate and metastasize.^9^ Currently, PD-L1 is a target for cancer therapy, and several monoclonal antibodies designed to bind the ectodomain of this transmembrane protein have been approved for clinical use.^10−13^ Thanks to recent advances in the characterization of PD-L1 biology, numerous small-molecule inhibitors have also been developed.^14,15^

An additional recent approach to inhibit the PD-1/PD-L1 axis relies on the use of the recombinant PD-1 ectodomain to address the PD-L1 protein on the surface of cancer cells.^16^ In fact, the formation of a complex between recombinant PD-1 and PD-L1 on cancer cells hampers the interaction of the latter with PD-1 exposed on T-cells and circumvents the main issue of the immune system suppression.^17,18^

It is noteworthy that recombinant PD-1 can also be used as a vector to target cancer cells overexpressing PD-L1 with probes, toxins, or therapeutic molecules. An advantage of this approach over the monoclonal antibodies is related to the possibility of using *Escherichia coli* as an expression system where this protein can be easily produced using straightforward manufacturing procedures. This strategy is also advantageous over using small molecules that are often difficult to modify without altering the affinity for the target. Particularly intriguing is the possibility of employing recombinant PD-1 as a carrier to address cancer cells with immune-stimulating agents. For example, the administration of l-rhamnose conjugated to proteins or peptides is known to induce an immune response through the generation of anti-rhamnose antibodies.^19,20^ These antibodies may be effective in activating macrophages and lymphocytes, thus, eliciting an immune response cascade.

For this purpose, a vast literature exists on the functionalization of endogenous or biocompatible macromolecules with *N*-hydroxysuccinimide (NHS)-activated rhamnosides as donors.^21,22^ Bioconjugation with NHS-activated molecules is indeed one of the most effective strategies to decorate or functionalize peptides or proteins under physiological conditions to modulate their solubility, bioavailability, or immunogenicity.

Recently, the high-affinity mutant of the N-terminal domain of PD-1, termed high-affinity consensus PD-1 (HAC-PD-1, hereafter), has been developed and successfully investigated for immunotherapy and PET imaging of cancers overexpressing PD-L1.^16^ A limitation of this effective mutant is, however, its limited versatility in terms of bioconjugation. As a matter of fact, conjugation of HAC-PD-1 with amine-reactively activated molecules is not feasible because two residues of lysine are located in the binding site for PD-L1. The functionalization of one or both of these residues would interfere with the binding properties of the mutant, likely preventing the interaction with PD-L1.

Here, we report on the design, biophysical characterization, and site-selective glycosylation of a new mutant of PD-1, namely, HACTR-PD-1. The HACTR-PD-1 mutant presents (i) a nanomolar affinity *vs* PD-L1, (ii) the N-terminal moiety as a unique amino group reacting with NHS-reagents, and (iii) suitable features for the development of new anti-PD-L1 proteins endowed with modular immunological activity. The site-selective functionalization of HACTR-PD-1 with polymers or rhamnosides as model glycans is herein described. The monofunctionalization of the mutant did not dampen its affinity vs PD-L1 and the immunomodulating properties of the derivatives obtained were investigated in vitro *vs* two different types of breast cancer (BC) cell lines. The HACTR-PD-1 rhamnosyl derivatives successfully prepared, namely, **1**-HACTR-PD-1 and **2**-HACTR-PD-1, are characterized by two different spacers and different types of glycosidic bonds used to link the rhamnosyl moiety to the mutant.

## Experimental Section

### Expression and Purification of the Human HACTR-PD-1 Mutant

*E. coli* BL21 (DE3) cells were transformed with the pET-28a (+) plasmid encoding the HACTR-PD-1 mutant (residues D26–R147, with the following mutations: V64H, L65V, N66V, Y68H, M70E, N74G, K78T, C93A, L122V, A125V, K131T, A132I, and K135R). In order to obtain uniformly isotopically enriched PD-1 [U–^15^N] and [U–^13^C, ^15^N], the cells were cultured in M9 minimal medium supplied with 1.1 g of ^15^N–NH_4_Cl or 1.1 g of ^15^N–NH_4_Cl and 3 g of ^13^C-glucose, respectively, 1 mL of 0.1 mg/mL solution of ampicillin, 1 mL of 1 mg/mL solution of thiamine, 1 mL of 1 mg/mL solution of biotin, 1 mmol·dm^–3^ MgSO_4_, and 0.3 mmol·dm^–3^ CaCl_2_; they were allowed to grow at 37 °C until OD600 reached 0.8 and then the overexpression was induced by 1 mmol·dm^–3^ isopropyl β-d-1-thiogalactopyranoside. The cells were further incubated at 37 °C overnight and then harvested by centrifugation at 6500 rpm (JA-10 Beckman Coulter) for 15 min at 4 °C. In all instances, the pellet was suspended at first in 50 mmol·dm^–3^ Tris-HCl, pH 8.0, 200 mmol·dm^–3^ NaCl, 10 mmol·dm^-3^ β-mercaptoethanol, and 10 mmol·dm^–3^ EDTA (50 mL per liter of culture) and sonicated for 30 s 10 times on ice at 4 °C. The lysate was centrifuged at 40,000 rpm (F15-6 × 100y Thermo Scientific) for 40 min, and the supernatant was discarded. The recovered pellet was resuspended in 50 mmol·dm^–3^ Tris-HCl, pH 8.0, 200 mmol·dm^–3^ NaCl, 10 mmol·dm^–3^ β-mercaptoethanol, and 6 mol·dm^–3^ guanidinium chloride (25 mL per liter of culture) and newly incubated overnight, at 4 °C, under magnetic stirring. Again, the suspension was centrifuged at 40,000 rpm (F15-6 × 100y Thermo Scientific) for 40 min. The pellet was discarded, whereas the supernatant containing the denatured protein solution was diluted in a refolding buffer containing 0.1 mol·dm^–3^ Tris-HCl, pH 8.5, 1 mol·dm^–3^ arginine, 0.25 mmol·dm^–3^ reduced glutathione, and 0.25 mmol·dm^–3^ oxidized glutathione. The solution was incubated at 4 °C under stirring for 12–18 h, clarified by passing through a 0.45 μm filter, and then dialyzed extensively against 10 mmol·dm^–3^ Tris-HCl, pH 8.0, and 20 mmol·dm^–3^ NaCl. The protein solution was concentrated with an Amicon Stirred Cell and then purified by size exclusion chromatography (SEC) using a HiLoad Superdex 26/60 75 pg (GE Healthcare) column previously equilibrated in 10 mmol·dm^–3^ Tris-HCl at pH 8.0 and 20 mmol·dm^–3^ NaCl.

### Functionalization of the HACTR-PD-1 Mutant with NHS-Activated PEG and Activated Rhamnose Derivatives **1** and **2**

Polyethylene glycol (PEG) conjugates of the HACTR-PD-1 mutant were obtained by reacting the protein with NHS ester derivatives of PEG-1000, PEG-5000 (Creative PEG Works), and rhamnosides (protein concentration around ∼1 mg/mL in 0.15 mol·dm^–3^ sodium phosphate buffer, pH 7.5, and reactive to a protein molar ratio of 15:1). After overnight incubation at room temperature with gentle stirring, the conjugates were purified from the unreacted fraction by SEC using a HiLoad Superdex 16/60 200 pg column and dialyzed on a 10 MW cut-off membrane against 0.15 mol·dm^–3^ sodium phosphate buffer, pH 7.5.

## Results and Discussion

The interaction of PD-1 on T-cells with PD-L1 on tumor cells reduces the T-cell function and dampens the antitumor immune response. Although beneficial under physiological conditions (autoimmunity control and modulation of harmful inflammations), in a tumoral scenario, such interactions promote tumor escape and progression. Impressive clinical results have been obtained by blocking the PD-1/PD-L1 interaction with monoclonal antibodies as specific inhibitors (immune checkpoint inhibitors) in advanced- and metastatic-stage cancers. Even though PD-1/PD-L1-neutralizing antibodies are considered the most promising drugs in cancer immunotherapy, the use of antibodies in some solid tumors^9^ has raised some concerns due to potential toxicity that may result from ADCC-mediated lysis of subsets of immune cells that express PD-L1.^23^ Therefore, current clinical trials are evaluating anti-PD-1/PD-L1 drugs for use in combination with other drugs or immune modulators.

### Design and Expression of the HACTR-PD-1 Mutant

Capitalizing on the potential of small proteins and their broad applicability in the modulation of the immune system, inspired by the already described HAC-PD-1 protein,^16^ an original set of PD-1 derivatives, where the two lysine residues K131 and K135 are replaced by nonreactive amino acids toward the NHS moiety, has been designed and screened in silico by performing docking calculations.

After structural analysis of the experimental HAC-PD-1/PD-L1 complex, three amino acids with different physical–chemical properties were taken into consideration to replace the two residual lysines present in the protein binding site: (i) a charged amino acid (Arg), (ii) a polar uncharged amino acid with a long side-chain (Gln), and (iii) a polar uncharged amino acid with a short side-chain (Thr). A computational study was performed by using the HADDOCK 2.2 web-portal^24^ to screen in silico the mutations on the stability of the complex with PD-L1. The complexes of PD-L1 with the two HAC-PD-1 mutants K131T/K135R and K131R/K135R showed the most favorable docking energies expressed in terms of HADDOCK-scores (see Table S1, Supporting Information). It is noteworthy that the stability of the two complexes is like that of the parent HAC-PD-1/PD-L1 adduct. The analysis of the calculated structural models revealed that in the K131R/K135R protein, the native small β-strand bearing the two mutated residues adopts a random coil shape. Conversely, this secondary structure element is well preserved in the K131T/K135R mutant (HACTR-PD-1). Therefore, this last mutant was selected for expression in *E. coli* to evaluate the correct folding and PD-L1-binding properties.

The spreading of the signals in the 1D ^1^H NMR and 2D ^1^H–^15^N HSQC spectra recorded on the HACTR-PD-1 protein is consistent with that of a well-structured protein. The 2D ^1^H–^15^N HSQC spectrum shows sharp and well-resolved signals. The backbone assignment of the protein, obtained from the analysis of triple-resonance spectra recorded on samples of [U–^13^C, ^15^N] HACTR-PD-1, allowed us to identify and characterize 114 spin systems over 122 total residues. The predictions of the secondary structure elements, obtained by TALOS+ analysis using the resonances of HACTR-PD-1 as input (Figure S1), indicate that the HACTR-PD-1 mutant has the same folding as the HAC-PD-1 protein. Concerning the protein partner, PD-L1, the assignment was already available in the BMRB (accession code 51169).^25^

**Figure 1:**
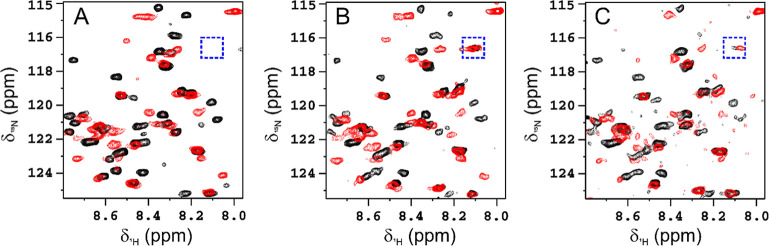
Region of 2D ^1^H–^15^N HSQC spectra of HACTR-PD-1/PD-L1 complexes superimposed with the corresponding references: (A) free HACTR-PD-1 (black) with respect to HACTR-PD-1 in the presence of PD-L1 (in 1:1 molar ratio, red); (B) HACTR-PD-1 conjugated with PEG 5 kDa (black) with respect to HACTR-PD-1 conjugated with PEG 5 kDa in the presence of PD-L1 (in 1:1 molar ratio, red); and (C) HACTR-PD-1 conjugated with l-rhamnose (black) with respect to HACTR-PD-1 conjugated with l-rhamnose in the presence of PD-L1 (in 1:1 molar ratio, red). The spectra were acquired on spectrometers operating at 900 (A), 950 (B), and 700 (C) MHz, ^1^H Larmor frequency, and 298 K. The spectra of the complexes were acquired with a higher number of scans than the reference spectra. The signal surrounded by the blue square is related to the new amide formed by conjugation with PEG5000 or the l-rhamnose derivative.

**Figure 2:**
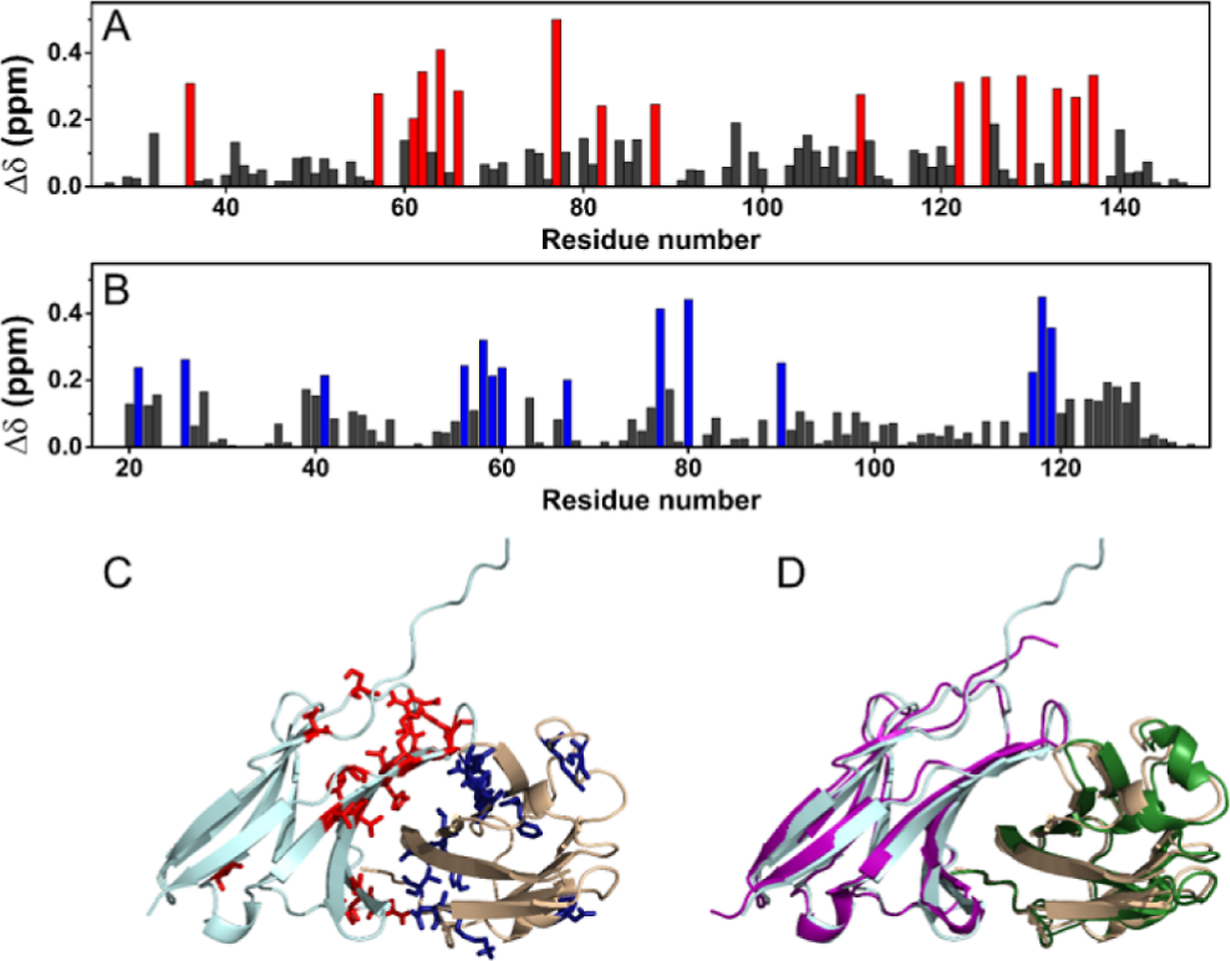
Chemical shift perturbation of HACTR-PD-1 in the presence of PD-L1 (A) and of PD-L1 in the presence of HACTR-PD-1 (B) (in a 1:1 molar ratio) evaluated using the Picasso Web server. The residues experiencing the largest perturbations have been highlighted in red and blue, respectively, and used as “active residues” in the HADDOCK calculation. (C) Model of the complex between HACTR-PD-1 (light cyan) and PD-L1 (wheat) with the lowest HADDOCK-score. The active residues have been highlighted as red and blue sticks, respectively. (D) Superimposition of the model of the complex evaluated with HADDOCK, where HACTR-PD-1 is in light cyan and PD-L1 is in wheat, with the X-ray structure (PDB code: 1IUS), where HAC-PD-1 is in purple and PD-L1 in green.

**Figure 3:**
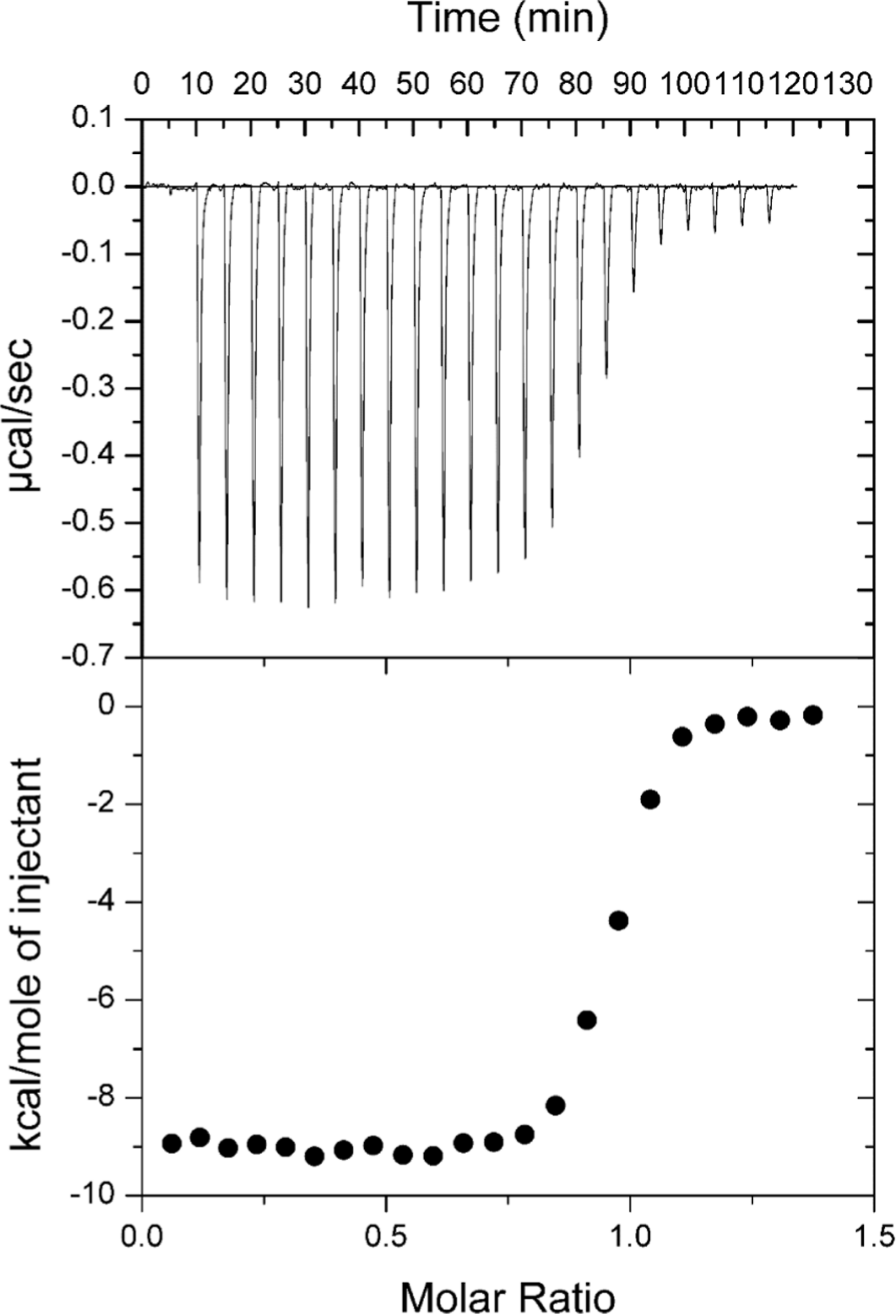
ITC data for the binding of the N-terminal domain of PD-L1 to HACTR-PD-1 in Tris buffer at pH 8.0 and 298 K. The thermogram is reported at the top, and the plot of the heat-released vs molar ratio is at the bottom of the panel. The data were fitted using a single binding site model.

Epitope mapping and qualitative information on the binding affinity were achieved by monitoring the evolution of the resonances of the ^15^N isotopically enriched HACTR-PD-1 in a 2D ^1^H–^15^N HSQC NMR spectrum upon the addition of increasing amounts of PD-L1 in natural abundance (Figure 1A and S2). In the spectra of HACTR-PD-1, the intensity of several signals decreases progressively, while new signals appear by increasing the concentration of PD-L1. These spectral changes fit with an interaction in the slow exchange regime on the NMR time scale. Complementary information was obtained by monitoring the evolution of the signals of the ^15^N isotopically enriched PD-L1 in a 2D ^1^H–^15^N HSQC NMR spectrum upon the addition of HACTR-PD-1 in natural abundance (Figure S3). This set of NMR data is consistent with a slow exchange regime on the NMR time scale previously observed and provides complementary information on the residues involved in the binding. To obtain the experimental restraints for the docking calculation of the adduct between HACTR-PD-1 and PD-L1, a prediction of the assignment of the resonances observed in the 2D ^1^H–^15^N HSQC NMR spectra of the complex and the related chemical shift perturbation set (Figure 2A,B) were generated by using the PICASSO Web server.^26^ In the program, the 2D ^1^H–^15^N HSQC reference spectra of free [U–^15^N] HACTR-PD-1 and free [U–^15^N] PD-L1 were compared to the spectra of [U–^15^N] HACTR-PD-1/PD-L1 and [U–^15^N] PD-L1/HACTR-PD-1 (in 1:1 molar ratio), respectively, to provide the assignment and the chemical shift perturbations on the two proteins in the complex. The residues experiencing the largest effects were imposed as “active residues” in the HADDOCK calculations (Figure 2C). The structural models of the mostly populated cluster (53 structures over 200) were also endowed with the lowest HADDOCK-score and were superimposable with the experimental X-ray structure of the complex (PDB code: 5IUS) available for the HAC-PD-1 protein (PyMOL rmsd of 0.875, Figure 2D). Then, the affinity of HACTR-PD-1 for PD-L1 was investigated by isothermal titration microcalorimetry (Figure 3). The titration was performed by adding HACTR-PD-1 to PD-L1 and provided a dissociation constant of 59 nmol·dm^–3^, which is in the good range for a drug candidate.

### Site-Selective Functionalization of the HACTR-PD-1 Mutant

Since the functionalization or bioconjugation of proteins involved in protein–protein complexes can lead to an undesired loss of affinity for the partner as functionalization can potentially mask the interaction surface, the effect of HACTR-PD-1 conjugation at the N-terminus was investigated by using two PEG chains of different sizes (1 and 5 kDa). These bulky polar chains have been chosen because they are often used to increase protein solubility, half-life in vivo, and renal clearance.^27−29^ Solutions of HACTR-PD-1 were thus reacted with a 15-fold molar excess of the two linear PEG chains properly activated as NHS ester derivatives. After chromatographic purification (see Supporting Information), samples of the two PEG-HACTR-PD-1 conjugates, obtained starting from ^15^N isotopically enriched proteins, were characterized by 2D ^1^H–^15^N HSQC NMR spectra.

The analysis of the spectra showed that the native folding is well preserved in the PEGylated proteins with only three residues at the N-terminus experiencing a large chemical shift variation. Also, the interaction between the two PEG-HACTR-PD-1 derivatives and PD-L1 is not negatively affected by the PEG hindering chains since the slow exchange regime and the distribution of the resonances observed in the 2D ^1^H–^15^N HSQC NMR spectra of the complexes are largely similar to those of the HACTR-PD-1 protein in complex with PD-L1 (Figure 1B and S4).

In keeping with this, to evaluate the potential of HACTR-PD-1 as an immunomodulating vector (see above), the mutant protein was conjugated to two selected rhamnopyranosides characterized by two different glycosidic linkages: the non-native, physiologically stable *C*- and the native *O*-glycosidic linkages.

### Synthesis of Activated Rhamnosides **1** and **2**

**Scheme 1:**
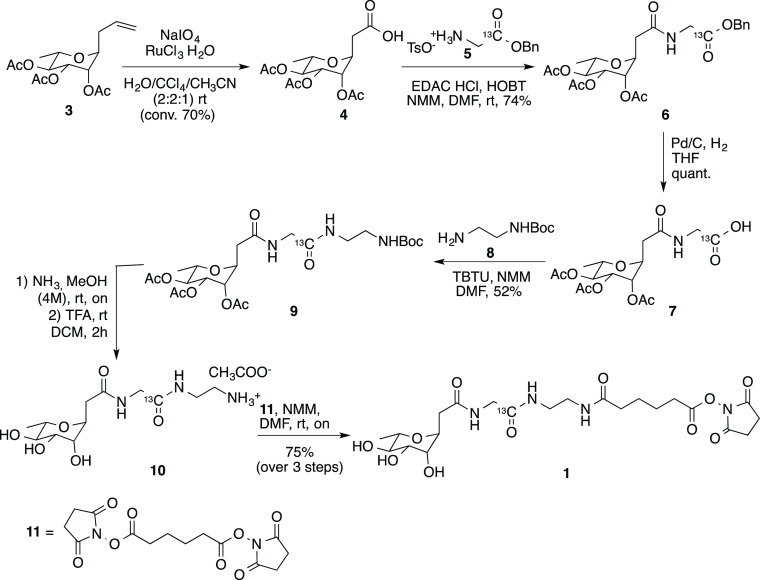
Synthesis of the α-*C*-l-rhamnoside **1**

**Scheme 2:**
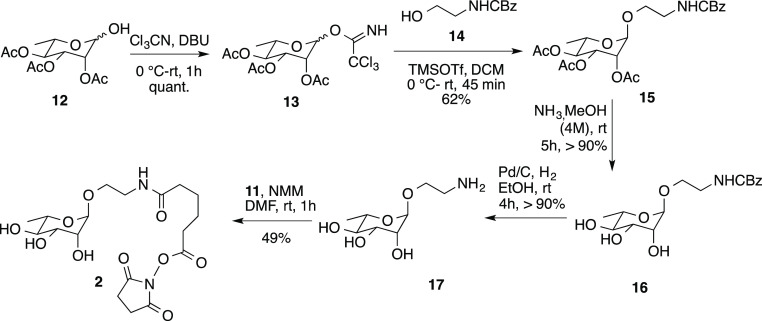
Synthesis of α-*O*-l-rhamnoside **2**

α-*C*-l-rhamnoside **1** and α-*O*-l-rhamnoside **2** were synthesized as displayed in Schemes 1 and 2.

In detail, *C*-rhamnoside **1** was obtained from the peracetylated C-allyl rhamnoside **3** as starting material, which was prepared as previously described.^30^ Upon oxidation with an excess of NaIO_4_ in the presence of RuCl_4_ as catalyst, compound **3** was transformed into carboxylic acid **4** (70% of conversion) which, in turn, was coupled with glycine ^13^C benzyl ester **5**. The coupling reaction was performed by using EDAC and HOBT as coupling reagents in the presence of NMM as a base and dry DMF as a solvent. The rhamnoside **6** so obtained (74%) was first transformed into the free carboxylic acid **7** (Pd/C, H_2_, THF, 2 h) and then reacted with the mono Boc-protected ethylenediamine **8** (with TBTU and NMM in dry DMF) to afford rhamnoside **9** (52%).

After the removal of the acetyl residues (NH_3_, 4 mol·dm^–3^ in MeOH, rt, overnight) and of the Boc protecting group (TFA, dry DCM, rt, 2 h), the crude **10** isolated as trifluoroacetic salt was reacted with **11** in the presence of NMM and dry DMF as the solvent. The desired rhamnosyl derivative **1** was thus obtained (75% over three steps), ready for the glycosylation of the PD-1 mutant (see Scheme 1 and Supporting Information).

Triacetyl rhamnoside **12**(31) was the starting material for the synthesis of rhamnoside **2**, which presents an *O*-glycosidic linkage and a shorted linker with respect to **1** (Scheme 2). Glycosylation of **12** with N-Boc protected ethanolamine **14** was performed relying on the trichloroacetimidate strategy by using trimethylsilyl triflate as a glycosidic promoter in dry DCM as a solvent. *O*-rhamnoside **15**, obtained as the α isomer (62%), was deacetylated with NH_3_ in MeOH (4 mol·dm^–3^, rt, 5 h, 16, >90%) and the benzyl protecting group removed by treatment with H_2_, catalytic Pd/C, and EtOH as a solvent (rt, 4 h, >90%) to afford the fully deprotected rhamnoside **17**. Compound **17** was finally reacted with linker **11** (see Scheme 1) to form the desired α-*O*-rhamnoside **2** suitably armed to glycosylate HACTR-PD-1 (Scheme 2 and Supporting Information).

**Figure 4:**
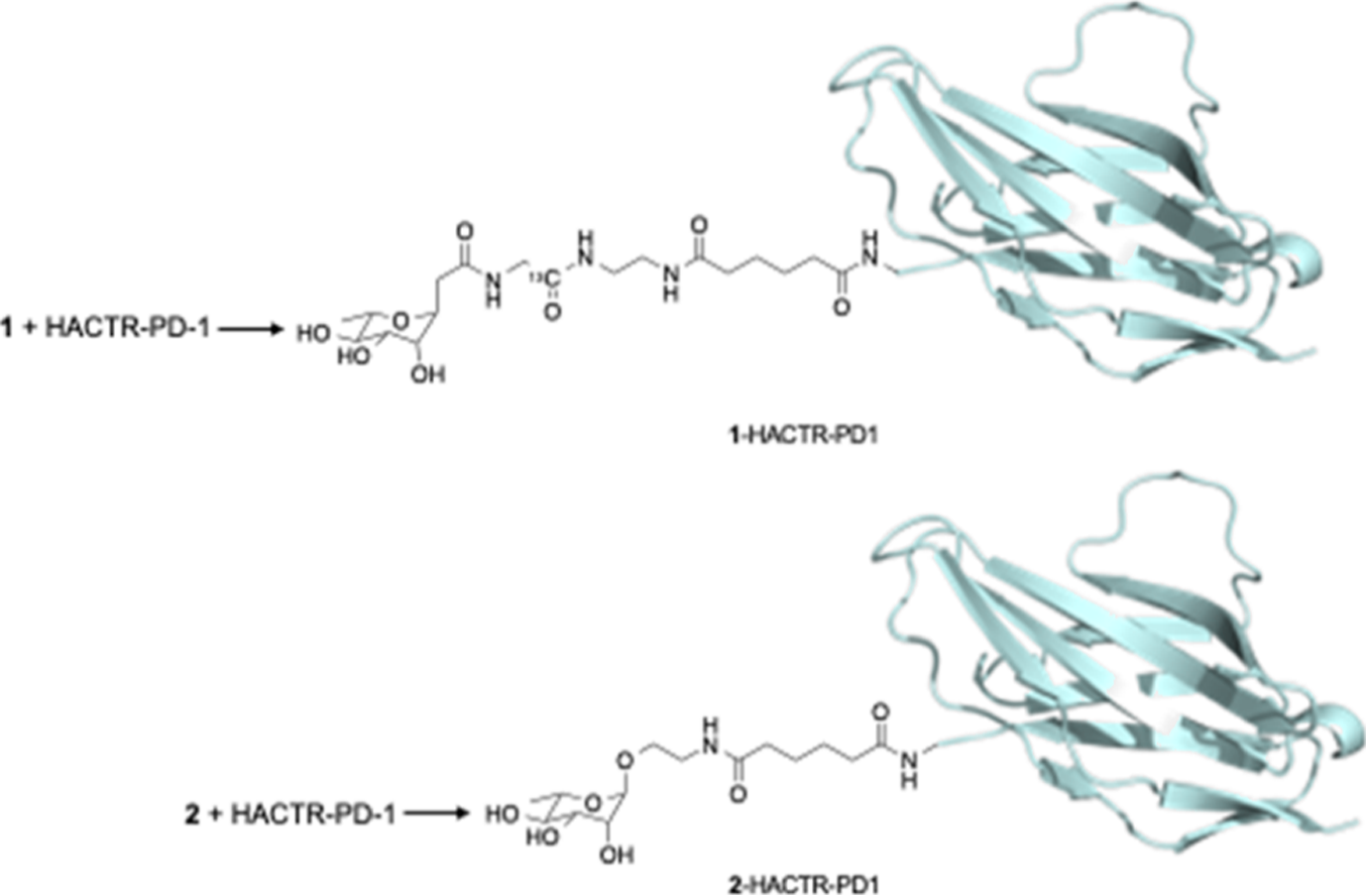
Rhamnosylated mutants **1**-HACTR-PD-1 and **2**-HACTR-PD-1.

The HACTR-PD-1 glycosylation was performed through two different linkers, properly activated as NHS-ester derivatives (Schemes 1 and 2), to afford the rhamnosyl mutants **1**-HACTR-PD1 and **2**-HACTR-PD1 (Figure 4). Both glycosylations proceeded smoothly at room temperature in phosphate buffer, and the two rhamnosyl mutants were obtained quantitatively after dialysis.

To confirm the data obtained for PEG-HACTR-PD-1, the rhamnosyl mutant **1**-HACTR-PD1 was screened by NMR for its binding properties *vs* those of PD-L1. A new signal corresponding to the functionalized N-terminus appeared in the 2D ^1^H–^15^N HSQC NMR spectrum, with a few signals corresponding to residues structurally closed to the N-terminus experiencing a sizable chemical shift variation. As previously observed for the PEGylated derivatives, the presence of l-rhamnose at the N-terminus of HACTR-PD-1 did not affect the interaction with PD-L1, which is still in the slow exchange regime on the NMR time scale (see Figure 1C and S6). This was indeed expected due to the smaller size of the rhamnoside with respect to the PEG. NMR analysis was used to verify that all the compounds were pure (>95% pure).

### Biological Tests

Finally, to investigate the biological profile of the new PD-1 mutant and of its l-rhamnosyl derivatives, a set of cell-based assays were carried out on wild-type (natural) PD-1, HACTR-PD-1, **1**-HACTR-PD-1, and **2**-HACTR-PD-1. The two rhamnosyl derivatives were screened to investigate the possible effects of the linker’s length and the glycosidic bond on cell tests.

PD-L1 has been established as a valuable biomarker for different types of cancer, and several studies have demonstrated that PD-L1 expression may be used to predict the outcome of the disease: patients with high PD-L1 expression have a significantly worse prognosis.^32^

BC is one of the most common tumors in women, and triple-negative breast cancer (TNBC) is the most aggressive BC, is difficult to treat, and has a poor prognosis. Antibodies targeting PD-1 or PD-L1 are currently considered a promising therapeutic strategy in TNBC.

MDA-MB-231 and MCF-7 cell lines represent valid study models for the evaluation of the activities of new compounds for BC therapy. In fact, these two cell lines embody a wide range of characteristics that are typically found in BC. MCF-7 is an ER-, PR-, and HER2-positive type of adenocarcinoma, while MDA-MB-231 cells stand for a triple-negative type of metastatic adenocarcinoma and are more aggressive than MCF-7 cells. Before testing the PD-1 variants’ activity on the selected tumor cell lines, we evaluated the expression of PD-L1 in each line. Flow cytometry analysis was thus performed, showing that PD-L1 is expressed on both cell lines (Figure S7). This result clearly confirms that both cell lines can be used to study the effect of the newly synthesized recombinant PD-1 proteins on restoring and/or modulating anti-tumor immune activity.

First, wild type PD-1, HACTR-PD-1, and **1**- and **2**-HACTR-PD-1 were tested to evaluate their immunomodulatory activity in a macrophage model. All proteins were active (see Figures S8–S10); glycosylated (**1**- and **2**-HACTR-PD-1) and not glycosylated (HACTR-PD-1) mutants displayed similar immunomodulatory activities in inducing M1 differentiation and TNF-α release, with a moderate but significant higher activity for glycosylated derivatives (Figure S10). M1 macrophages are pro-inflammatory cells involved in the recruitment and differentiation of other immune cells toward an anti-tumor phenotype. Of note, immune checkpoint inhibitors evoke a higher response when they are associated with other drugs able to stimulate the locked immune system.^33^ In this view, the ability of mutants (in particular of **1**- and **2**-HACTR-PD-1) to induce M1 differentiation can represent a valuable opportunity to educate macrophages toward a pro-inflammatory, anti-tumoral phenotype.

Afterward, the ability to overcome T cell inhibition mediated by PD-L1/PD-1 interaction was also investigated, starting from inhibited T cells’ regulatory and cytotoxic functions.^34^ Since both CD8 T and NK cells show cytotoxic activity against tumor cells and literature data suggest that both are affected by PD-L1/PD-1 binding, we performed a cytotoxic assay using peripheral blood mononuclear cells (PBMCs) that contain both populations. Cytotoxicity experiments were thus performed to verify the ability of PD-1 mutants to restore T and NK cell cytotoxic activity against tumor cells. Phytohemagglutinin (PHA)-stimulated PBMC were cocultured with CAM-stained tumor cells for 24 h, and the tumor cells’ death was analyzed by fluorescence activated cell sorting (FACS).

**Figure 5:**
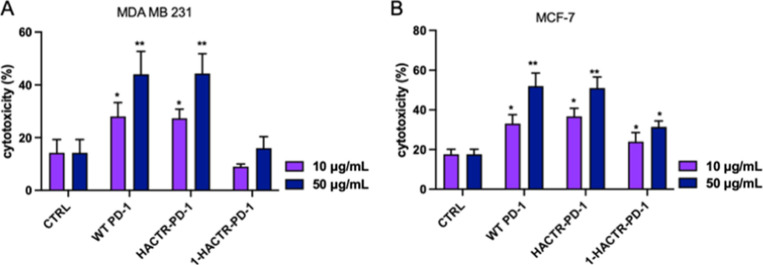
Effects of WT PD-1, HACTR-PD-1, and **1**-HACTR-PD-1 on T-cell-mediated BC cell cytotoxicity. CAM-labeled cancer cells were treated with 10 or 50 μg/mL of PD-1, HACTR-PD-1, or **1**-HACTR-PD-1 for 1 h and then incubated with PHA-stimulated PBMC. After 24 h of coculture, BC cells were harvested, and the level intensity of CAM was analyzed by FACS. (A) PBMC-mediated cytotoxicity against the MDA MB 231 cell line in the presence or absence of WT PD-1, HACTR-PD-1, or 1-HACTR-PD-1; (B) PBMC-mediated cytotoxicity against the MCF-7 cell line in the presence or absence of WT PD-1, HACTR-PD-1, or **1**-HACTR-PD-1. Results are expressed as the mean ± SEM of at least three independent experiments run in triplicate using PBMCs from three different donors. *p ≤ 0.05 natural/recombinant protein treated vs untreated cocultures; **p ≤ 0.01 natural/recombinant protein treated vs untreated cocultures. For data on **2**-HACTR-PD-1, see Figure S12.

As shown in Figure 5, WT PD-1 and HACTR-PD-1 were able to re-establish the CD8 T and NK cells’ cytotoxic activity in a concentration-dependent manner. As far as WT PD-1 and HACTR-PD-1 are concerned, similar data were collected for both MDA-MB-231 and MCF-7 cell line cocultures. Differently, for **1**-HACTR-PD-1, significant results were obtained in coculture with MCF-7 tumor cells. Comparable data were registered for **2**-HACTR-PD-1 (see Figure S11).

T helper cell functions are also affected by the PD-L1/PD-1 pathway.^35^ CD4 T helper cells are plastic cells that can differentiate toward the Th1 or Th2 phenotype depending on the milieu composition. In particular, Th1-polarized cells are pro-inflammatory cells defined by the production of interferon-γ (IFN-γ). These cells mediate cellular immunity through activation of pro-inflammatory macrophages and recruitment and promotion of the cytolytic activity of NK and CD8^+^ T cells, the main cells involved in the elimination of tumor cells. Moreover, IFN-γ can directly induce cell death through the binding of the IFN receptor and the induction of apoptosis. Another class of cells affected by the PD-L1/PD-1 pathway is represented by T regulatory (Treg) cells (CD4^+^CD25^+^). Tregs are a specialized T cell subpopulation involved in homeostasis and self-tolerance maintenance through immune response suppression. As known, Tregs inhibit T cell proliferation and cytokine production.^36^ Treg population is recruited to the tumor site and promotes the immune-suppressive microenvironment through different pathways mediated by both cellular and soluble components (i.e., CTLA-4/B7, TGF-β, and IL-10). IL-10 is an immunoregulatory cytokine that inhibits innate and acquired immune responses and has important immune-inhibitory activity. In the tumor context, the PD-L1/PD-1 pathway induces CD4^+^ conversion in highly suppressive T cells. In detail, PD-L1 engagement results in downregulation of PI3K/Akt/mTOR signaling that switches CD4 differentiation toward regulatory T cells (Treg), which in the tumor context take part in immunosuppression.^37^ Moreover, PD-1 signaling in CD4^+^ T cells reduces the PKC activation, which is essential for the activation of NF-κB transcription factors and responsible for the production of pro-inflammatory anti-tumoral cytokines like IFN-γ.^38^

Starting from these observations, we screened the ability of PD-1 mutants to restore IFN-γ production and inhibit the IL-10 release.

**Figure 6:**
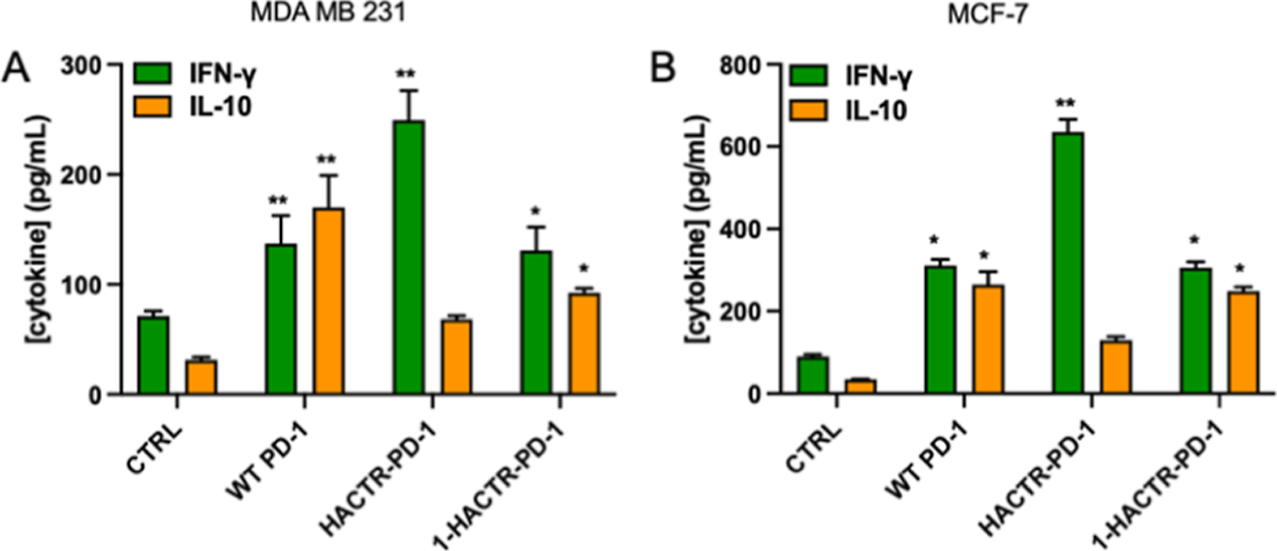
Effects of WT PD-1, HACTR-PD-1, and **1**-HACTR-PD-1 on T helper or Treg cytokine release. Cancer cells were treated with 10 μg/mL WT PD-1, HACTR-PD-1, or **1**-HACTR-PD-1 for 1 h and then incubated with PHA-stimulated PBMCs. After 48 h of cell culture, media were collected, and IFN-γ and IL-10 were measured by ELISA. (A) IFN-γ and IL-10 released by PBMCs cocultured with the MDA MB 231 cell line in the presence/absence of WT PD-1, HACTR-PD-1, or **1**-HACTR-PD-1; (B) IFN-γ and IL-10 released by PBMCs cocultured with the MCF-7 cell line in the presence or absence of WT PD-1, HACTR-PD-1, or **1**-HACTR-PD-1. Results are expressed as the mean ± SEM of at least three independent experiments run in triplicate using PBMCs from different donors. *p ≤ 0.05 natural/recombinant protein treated vs untreated cocultures; **p ≤ 0.01 natural or recombinant protein treated vs untreated cocultures. For data on **2**-HACTR-PD-1, see Figure S12.

PHA-stimulated PBMCs were cocultured with tumor cells for 48 h, and the levels of IFN-γ and IL-10 were assessed by ELISA. The presence of both tumor cell lines affected PBMC cytokine production with a reduction of the IFN-γ concentration and an increase in IL-10. In both MDA-MB-231 and MCF-7 cell line cocultures, WT PD-1, HACTR-PD-1, and **1**-HACTR-PD-1 were effective in increasing the IFN-γ levels (Figure 6). The highest IFN-γ release was observed upon HACTR-PD-1 treatment. In fact, HACTR-PD-1 in MDA-MB-231/PBMC coculture induces an increase in IFN-γ production of 250%, while WT PD-1 and **1**-HACTR-PD-1 induce an increase of 92 and 84%, respectively. When tested on MCF-7/PBMC coculture, all recombinant proteins induced higher IFN-γ production when compared with MDA-MB-231/PBMC-treated cells, even if the rates between the different treatments remained very similar. In all compound-treated cocultures, a significant increase in IL-10 production was observed (Figure 6), but this result was expected since with recombinant protein treatment, we have eliminated the PD-1/PD-L1 inhibitory effect on PHA-stimulated PBMCs. WT PD-1 and **1**-HACTR-PD-1 induced a higher level of IL-10, while HACTR-PD-1 induced a lower level in both coculture systems. Of note, when we analyzed the ratio between these cytokines that can be used as a rate of protumoral or anti-tumoral microenvironments, we observed that both HACTR-PD-1 and **1**-HACTR-PD-1 induced a clear increase in the IFN-γ/IL-10 ratio in both cell lines. These results suggest that both mutants participate in the establishment of an anti-tumor microenvironment able to sustain the anti-tumor immune responses.

**Figure 7:**
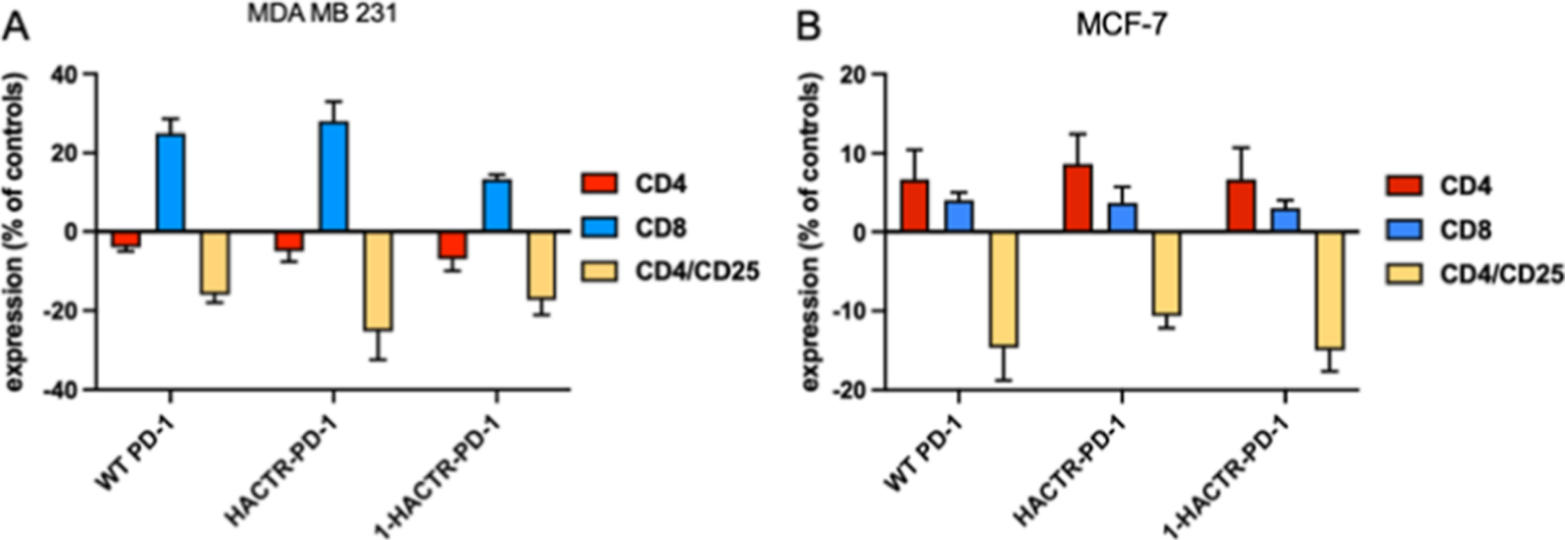
Effects of WT PD-1, HACTR-PD-1, and **1**-HACTR-PD-1 on the T subset percentage. Cancer cells were treated with 10 μg/mL PD-1, HACTR-PD-1, or **1**-HACTR-PD-1 for 1 h and then incubated with PHA-stimulated PBMCs. After 72 h of coculture, PBMCs were harvested, labeled with CD3, CD4, CD8, and CD25 monoclonal antibodies, and the percentage of CD4-, CD8-, and CD4–CD25-positive cells was evaluated by FACS. (A) CD4, CD8, and CD4–CD25 percentage after coculture with the MDA MB 231 cell line in the presence or absence of WT PD-1, HACTR-PD-1, or **1**-HACTR-PD-1; (B) CD4, CD8, and CD4–CD25 after coculture with the MCF-7 cell line in the presence or absence of WT PD-1, HACTR-PD-1, or **1**-HACTR-PD-1. Results are expressed as the mean ± SEM of at least three independent experiments run in triplicate using PBMCs from different donors. For data on **2**-HACTR-PD-1, see Figure S13.

To evaluate whether the tested mutants can induce changes in the lymphocyte subtype percentage after 72 h of tumor cell/PBMC coculture in the presence or absence of natural or recombinant proteins, PBMCs were harvested and labeled with anti-CD3, CD4, CD8, and CD25 antibodies and analyzed by FACS (Figure 7).

Although to a different extent, an increase in CD8 T cell percentage and a significant decrease in CD4/CD25 percentage (Figure 7) were detected for all mutants in PBMCs cocultured with both MDA-MB-231 and MCF-7 cell lines. An increase in CD4 was observed when PBMCs were cocultured with MCF-7 in the presence of recombinant proteins; conversely, when PBMCs were cocultured with MDA-MB-231, a slight but significant increase was observed for both CD4 and CD8 lymphocytes. All together, these data corroborate the properties of the compounds tested in mediating a desired anti-tumoral environment at the cellular level.

## Conclusions

In conclusion, in this article, we describe the design, expression, biophysical characterization, and site-selective glycosylation of the HACTR-PD-1 protein, a new mutant of the PD-1 ectodomain. The preservation of the folding state of the protein was proved by the minimal alteration of the 2D ^1^H–^15^N HSQC spectra and by the nanomolar affinity *vs* PD-L1 that was assessed by NMR spectroscopy and isothermal titration microcalorimetry as independent techniques. This new mutant, featuring the N-terminal moiety as a unique free amino group, was selectively functionalized with biocompatible moieties relevant for modulation of the PD-1 mutant in terms of solubility, clearance, and immunostimulant properties. The investigation of the biological profile of the new PD-1 mutant and of its l-rhamnosyl derivatives was carried out, and a set of cell-based assays was discussed. Rhamnoside tags have been selected as examples of known immunomodulators.^17−19^

The data collected clearly suggested that the HACTR-PD-1 mutant and its rhamnosyl derivatives reduced the anergy for all components of the T cells, with a resultant increase in proliferative and functional responses. Both HACTR-PD-1 and the rhamnosyl derivatives possess the ability to stimulate the innate immune system (in particular, macrophages) to set a pro-inflammatory environment essential to sustain an anti-tumoral immune response and to promote an anti-tumoral *milieu*, switching the IFN-γ/IL-10 ratio.

Rhamnosyl derivatives **1**-HACTR-PD-1 and **2**-HACTR-PD-1 showed similar activity in all tests, independent of the linker and glycosidic bond. Endowed with immunomodulating properties similar to, or in some cases, only slightly lower with respect to HACTR-PD-1, the site-selectively functionalized mutants, **1**-HACTR-PD-1 and **2**-HACTR-PD-1, thanks to the antigenic properties of rhamnose, represent the proof of concept for an unprecedented application of a PD-1 mutant as a ligand and also as a carrier with a panel of immunological activities. In perspective, the novel functionalized mutants, herein described, may pave the way for the development of new biologics to target the cancer cells overexpressing the PD-L1 receptor, not only by inhibiting the PD-1/PD-L1 interaction but also by exerting a more direct cytotoxic and immunological activity. On this forecast generation of molecules, a more in-depth investigation into ADME and immunological properties will be conducted.

## Supporting Information Available

Methods and materials, synthesis of rhamnosyl derivatives **1** and **2**, NMR measurements, and ITC titration (PDF)
