# Supplementary material for: Site-Selective Functionalized PD-1 Mutant for a Modular Immunological Activity against Cancer Cells
Source: Biomacromolecules. 2023 Oct 30;24(11):5428–37. doi: 10.1021/acs.biomac.3c00893 (PMC10646970; doi:10.1021/acs.biomac.3c00893)
Supplement: Supplementary file 1 — bm3c00893_si_001.pdf [file bm3c00893_si_001.pdf]

Supplementary Information

Site-selective functionalized PD-1 mutant for a modular immunological activity against cancer cells.

Silvia Fallarini,† Linda Cerofolini,‡,# Maria Salobehaj, ‡,# Domenico Rizzo, ‡,# Giulia Roxana Gheorghita, ‡,#,§ Giulia Licciardi,‡,# Daniela Eloisa Capialbi,‡ Valerio Zullo,‡ Andrea Sodini,‡ Cristina Nativi* ‡ and Marco Fragai* ‡,#.

† Department of Pharmaceutical Sciences, DSF, University of Piemonte Orientale, Largo Donegani 2 – 28100 Novara (NO), Italy

‡ Department of Chemistry, DICUS, University of Florence, via della Lastruccia 3,13 – 50019 Sesto F.no (FI), Italy

CeRM/CIRMMP, University of Florence, via L. Sacconi,6 – 50019 Sesto F.no (FI), Italy

§ Giotto Biotech, S.R.L, Via Madonna del piano 6, Sesto Fiorentino, Florence 50019, Italy.

Table of Content

Synthesis of compounds **3-10**	Pag.	S2-S4
Synthesis of compound **1**		S5
Synthesis of compounds **12-17**		S5-S7
Synthesis of compound **2**		S8
**Material and methods**		
Cell Culture		S9
Cell co-culture		S9
Proliferation assay		S9
Cytotoxicity assay		S9
THP1 cell culture and differentiation		S10
Flow cytometry		S10
ELISA assay		S10
Cell subset characterization		S10
Statistical analyzes		S10
Expression and purification of human wild-type PD-L1		S11
NMR Measurements		S11
NMR Titrations of the functionalized HACTR-PD-1 mutant with PD-L1		S11
HADDOCK Calculation		S12
ITC Titration of the HACTR-PD-1 mutant with PD-L1		S12
Table S1		S12
Figure S1		S13
Figure S2		S13
Figure S3		S14
Figure S4		S15
Figure S5		S15
Figure S6		S16
Figure S7		S16
Figure S8		S17
Figure S9		S18
Figure S10		S18
Figure S11		S19
Figure S12		S20
Figure S13		S20
Figure S14		S21
**References**		S21

Synthesis of compound 3:

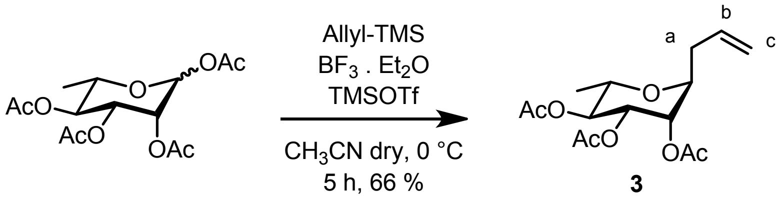

Allyl-TMS (690 µL, 4.34 mmol), $BF_3 \cdot Et_2O$ (1.2 mL, 4.34 mmol) and TMSOTf (20 µL, 0.11 mmol) were added to a solution of peracetylated L-rhamnose (720 mg, 2.17 mmol) in dry CH_3CN (4 mL) at 0°C. The reaction mixture was stirred at 0°C for 5 hours, then neutralized with Et_2O (5 mL) and $NaHCO_3$ (s.s., 10 mL), diluted with EtOAc, washed with H_2O (2x) and brine (2x). The organic layer was dried over anhydrous Na_2SO_4 and the solvent removed under vacuum. The crude mixture was purified by flash chromatography on silica gel (PE/EtOAc : 8/2) to yield **3** as a colourless oil (450 mg, 66 % yield).

MW: ($C_{15}H_{22}O_7$) 314.33 g/mol.
ESI-MS: *m/z* (%) 337 (100) $[M+Na]^+$.
^{1}H NMR: (500 MHz, $CDCl_3$) δ: 5.79 (ddt, $J_{b\text{-}c\ trans}$ = 17.1 Hz, $J_{b\text{-}c\ cis}$ = 10.1 Hz, $J_{b\text{-}a}$ = 6.95 Hz, 1H, H-b), 5.16 – 5.12 (m, 4H, H-3 + H-2 + H-c), 5.06 – 5.01 (m, 1H, H-4), 3.99 – 3.93 (m, 1H, H-1), 3.78 (dq, $J_{5\text{-}4}$ = 8.5 Hz, $J_{5\text{-}6}$ = 6.3 Hz 1H, H-5), 2.58 – 2.51 (m, 1H, 1H of H-a) 2.45 – 2.38 (m, 1H, 1H of H-a), 2.12 (s, 3H, Ac), 2.06 (s, 3H, Ac), 2.01 (s, 3H, Ac), 1.23 (d, $J_{6\text{-}5}$ = 6.3 Hz, 3H, H-6) ppm.
^{13}C NMR: (125 MHz, $CDCl_3$) δ: 170.3 (C_q, CO), 170.1 (C_q, CO), 169.9 (C_q, CO), 132.9 (CH, C-b), 118.2 (CH_2, C-c), 74.4 (CH, C-1), 71.5 (CH, C-4), 70.4 (CH, C-3), 69.1 (CH, C-2), 68.2 (CH, C-5), 33.7 (CH_2, C-a), 20.9 (CH_3, Ac), 20.8 (CH_3, Ac), 20.7 (CH_3, Ac), 17.6 (CH_3, C-6) ppm.

Synthesis of compound 4:

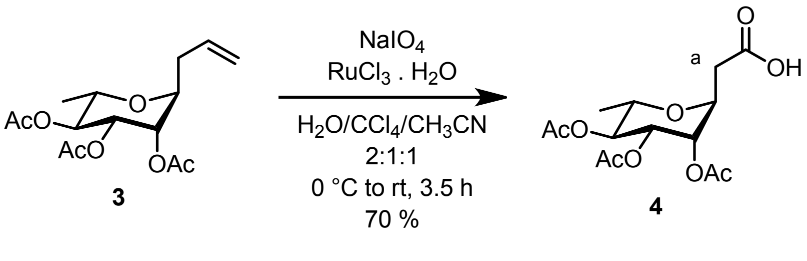

$NaIO_4$ (1.14 g, 5.34 mmol) and $RuCl_3 \cdot H_2O$ (12 mg, 0.053 mmol) were added to a solution of **3** (420 mg, 1.33 mmol) in the mixture of solvents $H_2O:CCl_4:CH_3CN$ = 2:1:1 (14 mL) at 0°C. The reaction mixture was stirred at room temperature for 3.5 hours, after which it was filtered on #4 frit filtered on a Celite® pad, washing with H_2O (2x) and $NaHCO_3$ (s.s. 2x). The mixture of solvents was transferred in a separating funnel and the aqueous layer was washed with CH_2Cl_2 (3 times). The combined organic phases were extracted with H_2O (2x) and $NaHCO_3$ (s.s. 2x). The aqueous layers were pulled together, acidified to pH=4 by addition of conc. HCl, then reextracted with CH_2Cl_2 (3x). The organic phase was dried over Na_2SO_4 and solvents were removed in vacuo to obtain 300 mg of crude **4** that was progressed to the next step without further purification.

MW: ($C_{14}H_{20}O_9$) 332.31 g/mol.
ESI-MS: *m/z* (%) 331 (100) $[M - H]^-$.
^{1}H NMR: (500 MHz, $CDCl_3$) δ: 5.23 (dd, 1H, $J_{2\text{-}3}$ = 3.4 Hz, $J_{3\text{-}4}$ = 7.5 Hz, H-3), 5.17 (dd, 1H, $J_{1\text{-}2}$ = 4.9 Hz, $J_{2\text{-}3}$ = 3.4 Hz, H-2), 5.00 (dd, 1H, $J_{4\text{-}3}$ = 7.5 Hz, $J_{4\text{-}5}$ = 6.5 Hz, H-4), 4.48 – 4.42 (m, 1H, H-1), 3.96 – 3.89 (aquint, 1H, J = 6.5 Hz, H-5), 2.82 – 2.76 (A part of an ABX system, $J_{a\text{-}b}$ = 15.5 Hz, $J_{a\text{-}x}$ = 9.6 Hz, 1H, 1H of

H-a), 2.70 – 2.66 (B part of an ABX system, 1H, $J_{b\text{-}a}$ = 15.5 Hz, $J_{b\text{-}x}$ = 4.6 Hz, one H of H-a), 2.12 (s, 3H, Ac), 2.11 (s, 3H, Ac), 2.08 (s, 3H, Ac), 1.35 (d, $J_{6\text{-}5}$ = 6.6 Hz, 3H, C-6) ppm.
^{13}C NMR: (125 MHz, $CDCl_3$) δ: 174.5 (C_q, CO), 170.1 (C_q, CO), 169.9 (C_q, CO), 169.9 (C_q, CO), 71.4 (CH, C-4), 69.8 (CH, C-5), 69.7 (CH, C-2), 69.3 (CH, C-1), 68.5 (CH, C-3), 35.3 (CH_2, C-a), 20.9 (CH_3, Ac), 20.8 (CH_3, Ac), 20.7 (CH_3, Ac), 16.9 (CH_3, C-6) ppm.

Synthesis of compound 6:

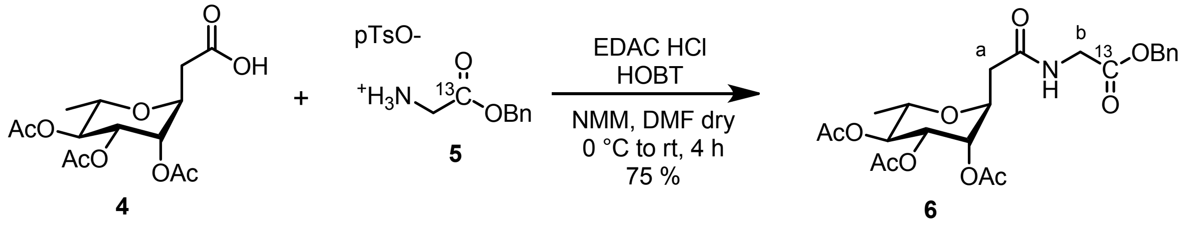

To a solution of **4** (280 mg, 0.84 mmol) in dry DMF (3.8 mL), EDAC·HCl (315 mg, 2.02 mmol) and HOBT (250 mg, 1.85 mmol) were added at 0 °C (solution A). The solution was stirred at 0 °C for 30 minutes. To a solution of **5** (345 mg, 1.01 mmol) in dry DMF (6.6 mL), NMM (333 µL, 3.03 mmol) was added at 0 °C (solution B). The solution was stirred at 0 °C for 20 minutes. Solution B was then added to solution A and the reaction mixture stirred at 0 °C for 40 minutes and at room temperature for 3 hours. The mixture was then diluted with EtOAc and washed with HCl 3% (3x), $NaHCO_3$ (s.s., 3x) and brine (3x). The organic layer was dried over anhydrous Na_2SO_4 and the organic solvent removed under vacuum. The crude was purified by flash chromatography on silica gel (PE/EtOAc : 45/55) to yield compound **6** as a colourless oil (360 mg, 75 % yield).

MW: ($C_{22}{}^{13}CH_{29}NO_{10}$) 480.46 g/mol.
ESI-MS: *m/z* (%) 480 (100) $[M + Na]^+$.
^{1}H NMR: (500 MHz, $CDCl_3$) δ: 7.42 – 7.33 (m, 5H, Ph), 6.74 (bs, 1H, NH), 5.24 (dd, $J_{3\text{-}4}$ = 6.6 Hz, $J_{3\text{-}2}$ = 3.4 Hz, 1H, H-3), 5.20 (d, J = 3.2 Hz, 2H, CH_2-Ph) 5.11 (dd, $J_{2\text{-}1}$ = 6.1 Hz, $J_{2\text{-}3}$ = 3.4 Hz, 1H, H-2), 4.95 (dd, $J_{4\text{-}3}$ = 6.6 Hz, $J_{4\text{-}5}$ = 5.3 Hz, 1H, H-4), 4.41 (ddd, $J_{1\text{-}Ha}$ = 9.8 Hz, $J_{1\text{-}2}$ = 6.1 Hz, $J_{1\text{-}Ha}$ = 3.7 Hz, 1H, H-1), 4.14 – 4.05 (m, 2H, H-b), 3.99 – 3.93 (m, 1H, H-5), 2.66 – 2.60 (A part of ABX system, $J_{a\text{-}b}$ = 15.5 Hz, $J_{a\text{-}x}$ = 9.8 Hz, 1H, 1H of H-a), 2.58 – 2.53 (B part of ABX system, $J_{b\text{-}a}$ = 15.5 Hz, $J_{b\text{-}x}$ = 3.7 Hz, 1H, 1H of H-a), 2.10 (s, 3H, Ac), 2.08 (s, 3H, Ac), 2.08 (s, 3H, Ac), 1.38 (d, $J_{6\text{-}5}$ = 6.6 Hz, 3H, H-6) ppm.
^{13}C NMR: (125 MHz, $CDCl_3$) δ: 169.9 (C_q, CO), 169.8 (C_q, CO), 169.7 (C_q, CO), 169.7 ($^{13}C_q$, CO), 169.4 (C_q, CO), 135.1, 128.7, 128.6, 128.4, 71.5 (CH, C-4), 70.3 (CH, C-5), 69.5 (CH, C-2), 68.7 (CH, C-3), 68.2 (CH, C-1), 67.2 (CH_2, OBn), 41.5 (d, $J_{c\text{-}c}$ = 61.8 Hz, CH_2, C-b), 36.8 (CH_2, C-a), 20.9 (CH_3, Ac), 20.8 (CH_3, Ac), 20.8 (CH_3, Ac), 16.7 (CH_3,C-6) ppm.

Synthesis of compound 9:

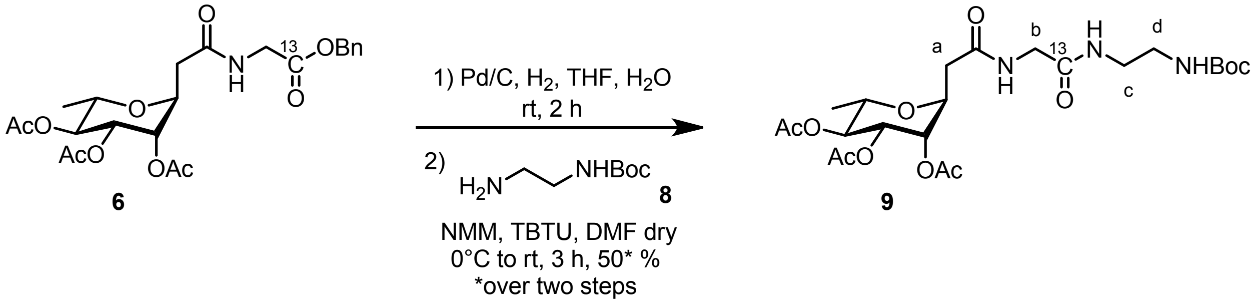

Pd/C (66 mg, 106.42 mmol) was added to a solution of **6** (270 mg, 0.56 mmol) in THF (7 mL) and H_2O (30 µL). The suspension was stirred at room temperature under H_2 atmosphere for 2 hours. The mixture was then filtered on a Celite® pad, washing with THF and the solvent was removed under vacuo. The crude compound was then resolubilized in DMF (2.7 mL), then TBTU (328 mg, 1.02 mmol), NMM (156 mg, 1.53 mmol) and compound **8** (106 mg, 0.66 mmol) were added at 0°C. The mixture was stirred at room temperature for 3 hours then diluted with EtOAc and washed with HCl 3% (3x), $NaHCO_3$ (s.s. 3x) and brine (3x). The organic layer was dried over Na_2SO_4 and the organic solvent evaporated to dryness. The crude was purified via flash chromatography on silica gel (CH_2Cl_2:CH_3OH / 95:5), to yield compound **9** as a pale yellow oil (140 mg, 50 % yield over two steps).

MW: ($C_{22}{}^{13}CH_{37}N_3O_{11}$) 532.55 g/mol.

ESI-MS: *m/z* (%) 555 (100) $[M + Na]^+$.

^{1}H NMR: (500 MHz, $CDCl_3$) δ: 7.34 (bs, 1H, NH), 7.16-7.10 (m, 1H, NH), 5.28 (bs, 1H, NH), 5.19 (dd, $J_{3\text{-}4}$ = 6.6 Hz, $J_{3\text{-}2}$ = 3.4 Hz, 1H, H-3), 5.14 (dd, $J_{2\text{-}1}$ = 6.1 Hz, $J_{2\text{-}3}$ = 3.4 Hz, 1H, H-2), 4.97 (dd, $J_{4\text{-}3}$ = 6.6 Hz, $J_{4\text{-}5}$ = 5.3 Hz, 1H, H-4), 4.43 – 4.37 (m, 1H, H-1), 3.95 – 3.88 (m, 3H, H-5 + H-b), 3.39 – 3.32 (m, 2H, H-c), 3.27 – 3.21 (m, 2H, H-d), 2.74 – 2.67 (A part of an ABX system, $J_{a\text{-}b}$ = 15.0 Hz, $J_{a\text{-}x}$ = 9.6 Hz, 1H, 1H of H-a), 2.61 – 2.53 (B part of ABX system, $J_{b\text{-}a}$ = 15.0 Hz, $J_{b\text{-}x}$ = 4.7 Hz, 1H, 1H of H-a), 2.08 (s, 3H, Ac), 2.07 (s, 3H, Ac), 2.03 (s, 3H, Ac), 1.41 (s, 9H, tBu), 1.31 (d, $J_{6\text{-}5}$ = 6.6 Hz, 3H, H-6) ppm.

^{13}C NMR: (125 MHz, $CDCl_3$) δ: 170.4 (C_q, CO), 170.1 (C_q, CO), 169.9 (C_q, CO), 169.9 (C_q, CO), 169.8 (C_q, CO), 169.3 ($^{13}C_q$, CO), 79.7 (C_q), 71.4 (CH, C-4), 69.9 (CH, C-1), 69.7 (CH, C-5), 68.7 (CH, C-2), 68.5 (CH, C-3), 43.15 (d, $J_{c\text{-}c}$ = 52.3 Hz, CH_2, C-b), 40.6 (CH_2, C-c), 40.2 (CH_2, C-d), 36.7 (CH_2, C-a), 28.4 ($3CH_3$, *t*Bu), 20.9 (CH_3, Ac), 20.8 (CH_3, Ac), 20.7 (CH_3, Ac), 17.0 (CH_3, C-6) ppm.

Synthesis of compound 10:

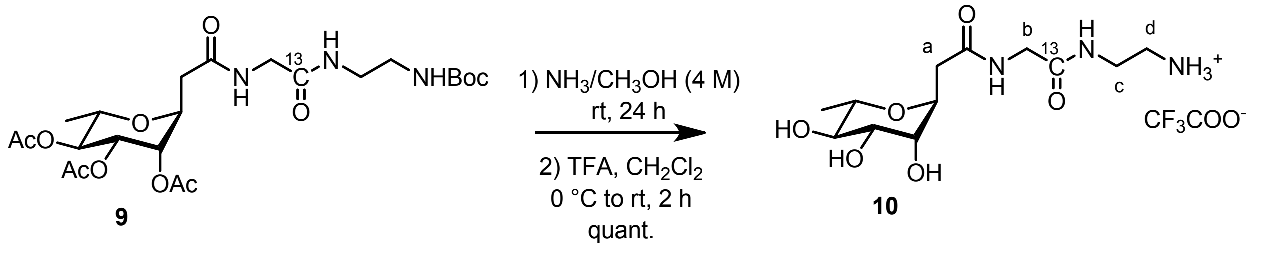

Compound **9** (120 mg, 0.22 mmol) was dissolved in 0.5 mL of a 4 M solution of NH_3 in CH_3OH. The reaction mixture was stirred at room temperature for 24 hours then evaporated to dryness. The crude compound was then resuspended in anhydrous CH_2Cl_2 (1.6 mL) and TFA (120 µL, 1.56 mmol) was added dropwise at 0 °C. The solution was stirred at room temperature for 2 hours then it was evaporated to dryness to give crude **10** (140 mg) which was used without further purification.

MW: ($C_{13}{}^{13}CH_{24}F_3N_3O_8$) 420.35 g/mol.
ESI-MS: *m/z* (%) 307 (100) $[M + H]^+$.
^{1}H NMR: (500 MHz, CD_3OD) δ: 4.31 (ddd, $J_{1\text{-}Ha}$ = 9.1 Hz, $J_{1\text{-}2}$ = 5.4 Hz, $J_{1\text{-}Ha}$ = 3.7 Hz, 1H, H-1), 3.90 – 3.85 (m, 2H, H-b), 3.80 (at, $J_{2\text{-}H}$ = 3.6 Hz, 1H, H-2), 3.69 (dd, 1H, $J_{3\text{-}4}$ = 6.6 Hz, $J_{3\text{-}2}$ = 3.4 Hz, H-3), 3.67 – 3.61 (m, 1H, H-5), 3.53 – 3.47 (m, 3H, H-4+H-c), 3.08 (t, $J_{d\text{-}c}$ = 5.8 Hz, 2H, H-d), 2.75 – 2.69 (A part of an ABX system, $J_{a\text{-}b}$ = 14.5 Hz, $J_{a\text{-}x}$ = 9.1 Hz, 1H, 1H of H-a), 2.60 – 2.54 (B part of an ABX system, $J_{b\text{-}a}$ = 14.5 Hz, $J_{b\text{-}x}$ = 5.4 Hz, 1H, 1H of H-a), 1.30 (d, $J_{6\text{-}5}$ = 6.3 Hz, 3H, H-6) ppm.

^{13}C NMR: (125 MHz, CD_3OD) δ: 172.3 (C_q, CO), 170.4 ($^{13}C_q$, CO), 72.7 (CH, C-1), 71.1 (CH, C-4), 71.1 (CH, C-5), 71.1 (CH, C-3), 70.8 (CH, C-2), 42.4 (d, $J_{c\text{-}c}$ = 52.6 Hz, CH_2, C-b), 39.6 (CH_2, C-d), 36.6 (CH_2, C-c), 36.2 (CH_2, C-a), 16.7 (CH_3, C-6) ppm.

Synthesis of compound 1:

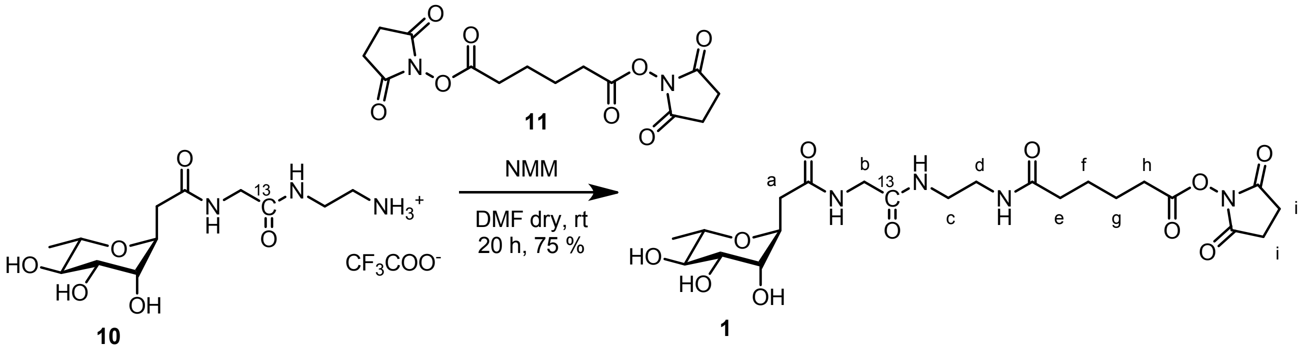

NMM (58 mg, 0.58 mmol) was added to a solution of **10** (115 mg, 0.15 mmol) in dry DMF (1.6 mL). After 20 minutes, compound **11** (220 mg, 0.64 mmol) [1] was added. The reaction mixture was stirred at room temperature for 20 hours then the suspension obtained was filtered on #4 frit and washed with DMF (2x). The solid (unreacted linker **11**) was discarded, while the organic phase was evaporated to dryness. The crude was purified by precipitation with EtOAc, the precipitate was filtered on #4 frit, washed with EtOAc (2x) and dried under vacuum affording the desired compound **1** (65 mg, 70 % yield over three steps).

MW: ($C_{21}{}^{13}CH_{34}N_4O_{11}$) 531.52 g/mol.
ESI-MS: *m/z* (%) 554 (100) $[M + Na]^+$, 570 (100) $[M + K]^+$.
$[\alpha]_D^{22}$ = +2.9 (c=0.1 in CH_3OH).
^{1}H NMR: (500 MHz, CD_3OD) δ: 4.31 (ddd, $J_{1\text{-}Ha}$ 9.1 Hz, $J_{1\text{-}2}$ 5.3 Hz, $J_{1\text{-}Ha}$ 3.7 Hz, 1H, H-1), 3.88 – 3.82 (m, 2H, H-b), 3.79 (at, $J_{2\text{-}H}$ = 3.6 Hz, 1H, H-2) 3.69 (dd, 1H, $J_{3\text{-}2}$ = 3.4 Hz, $J_{3\text{-}4}$ = 6.6 Hz, H-3), 3.67 – 3.61 (m, 1H, H-5) 3.49 (t, $J_{4\text{-}3}$ = 6.6 Hz, 1H, H-4), 3.31 (m, 4H, H-c, H-d), 2.86 (s, 4H, H-i+H-l), 2.71 – 2.76 (m, 3H, H-e + 1H of H-a), 2.60 – 2.54 (B part of an ABX system, $J_{b\text{-}a}$ 14.5 Hz, $J_{b\text{-}x}$ 5.5 Hz, 1H, H-a) 2.28 – 2.24 (m, 2H, H-h), 1.81 – 1.69 (m, 4H, H-f + H-g), 1.30 (d, $J_{6\text{-}5}$ = 6.3 Hz, 3H, CH_3-6) ppm.
^{13}C NMR: (125 MHz, CD_3OD) δ: 174.6 (C_q, CO), 172.3 (C_q, CO), 170.6 (C_q, CO), 170.5 ($^{13}C_q$, CO), 168.7 (C_q, CO), 72.7 (CH, C-1), 71.0 (CH, C-4), 71.0 (CH, C-5), 71.0 (CH, C-3), 70.7 (CH, C-2), 42.5 (d, $J_{c\text{-}c}$ = 52.6 Hz, CH_2, C-b), 38.8 (CH_2, C-d), 38.4 (CH_2, C-c), 36.30 (CH_2, C-a), 35.0 (CH_2, C-h), 29.8 (CH_2, C-e), 24.8 (CH_2, C-i + C-l), 24.5 (CH_2, C-f), 23.8 (CH_2, C-g), 16.6 (CH_3, C-6) ppm.

Synthesis of compound 12:

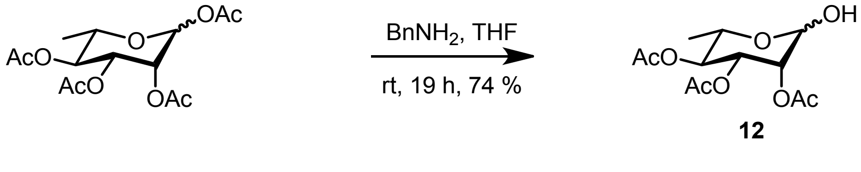

Benzylamine (1.6 mL, 14.5 mmol) was added to a solution of peracetylated rhamnose (969 mg, 2.9 mmol) in THF (16 mL). The mixture was stirred at room temperature for 19 hours after which a 1 M solution of HCl (60 mL) was added and stirred for 30 minutes. The water phase was then extracted with CH_2Cl_2 (x4), the organic layer was dried over anhydrous Na_2SO_4 and concentrated under vacuum. The crude mixture (1.15 g)

was purified by flash chromatography on silica gel (PE/EtOAc : 6/4), yielding **12** as a white solid (623 mg, 74 % yield, α anomer 90 % - β anomer 10 %).

Characterization of α anomer:
M.W.: ($C_{26}H_{38}O_{17}$) 622.57 g/mol.
^{1}H NMR: (α anomer) (500 MHz, $CDCl_3$) δ: 5.35 (dd, $J_{3\text{-}4}$ = 10.0 Hz, $J_{3\text{-}2}$ = 3.4 Hz, 1H, H-3), 5.25 (dd, $J_{2\text{-}3}$ = 3.4 Hz, $J_{2\text{-}1}$ = 1.8 Hz, 1H, H-2), 5.14 (dd, $J_{1\text{-}OH}$ = 3.9 Hz, $J_{1\text{-}2}$ = 1.8 Hz, 1H, H-1), 5.06 (t, J = 9.9 Hz, 1H, H-4), 4.12 (dq, $J_{5\text{-}4}$ = 9.9 Hz, $J_{5\text{-}6}$ = 6.3 Hz, 1H, H-5), 3.56 (d, $J_{OH\text{-}1}$ = 3.9 Hz, 1H, OH), 2.14 (s, 3H, Ac), 2.04 (s, 3H, Ac), 1.98 (s, 3H, Ac), 1.20 (d, $J_{6\text{-}5}$ = 6.3 Hz, 3H, H-6) ppm.
^{13}C NMR: (α anomer) (125 MHz, $CDCl_3$) δ: 170.5 (C_q, CO), 170.3 (C_q, CO), 170.3 (C_q, CO), 92.2 (CH, C-1), 71.3 (CH, C-4), 70.4 (CH, C-2), 69.0 (CH, C-3), 66.5 (CH, C-5), 21.1 (CH_3, Ac), 20.9 (CH_3, Ac), 20.9 (CH_3, Ac), 17.6 (CH_3, C-6) ppm.

Synthesis of compound 13:

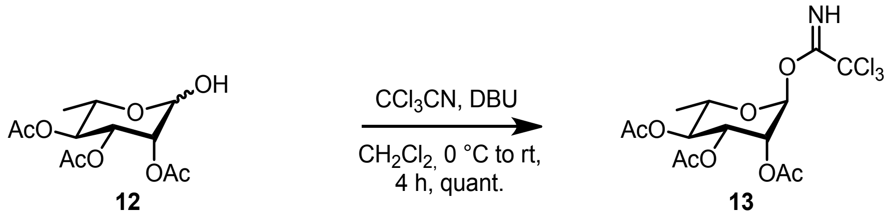

12 **13**

To a solution of **12** (393 mg, 1.35 mmol) in dry CH_2Cl_2 (15 mL), trichloroacetonitrile (1.0 mL, 10.0 mmol) and DBU (100 µL, 0.67 mmol) were added at 0 °C under N_2 atmosphere. The mixture was stirred at room temperature for 4 hours, then the mixture was diluted with CH_2Cl_2 (30 mL) and washed with NH_4Cl (s.s. x3), H_2O (x2) and eventually brine (x1). The organic phase was dried over Na_2SO_4 and concentrated *in vacuo* to give the crude **13** as a brown foam (679 mg, quant. yield) which was used without further purification.

M.W.: ($C_{14}H_{18}Cl_3NO_8$) 434.65 g/mol.
^{1}H NMR: (500 MHz, $CDCl_3$) δ: 8.72 (s, 1H, NH), 6.20 (d, $J_{1\text{-}2}$ = 1.9 Hz, 1H, H-1), 5.45 (dd, $J_{2\text{-}3}$ = 3.5 Hz, $J_{2\text{-}1}$ = 2.0 Hz, 1H, H-2), 5.36 (dd, $J_{3\text{-}4}$ = 10.2 Hz, $J_{3\text{-}2}$ = 3.5 Hz, 1H, H-3), 5.17 (t, $J_{4\text{-}H}$ = 10.0 Hz, 1H, H-4), 4.09 (dq, $J_{5\text{-}4}$ = 10.0 Hz, $J_{5\text{-}6}$ = 6.2 Hz, 1H, H-5), 2.18 (s, 3H, Ac), 2.07 (s, 3H, Ac), 2.00 (s, 3H, Ac), 1.27 (d, $J_{6\text{-}5}$ = 6.2 Hz, 3H, H-6) ppm.

Synthesis of compound 15:

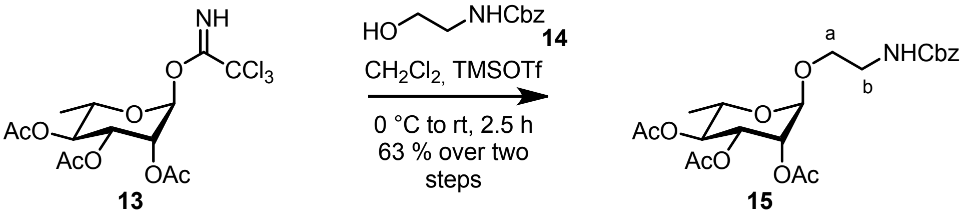

13 **15**

To a solution of **13** (679 mg, 1.35 mmol) and Z-ethanolamine **14** (401 mg, 2.05 mmol) in dry CH_2Cl_2 (10 mL), TMSOTf (60 µL, 0.33 mmol) was added at 0 °C under a N_2 atmosphere. The mixture was stirred at room temperature for 2.5 hours, then the reaction mixture was diluted with CH_2Cl_2 (30 mL) and washed with H_2O

(x3) and brine (x1). The organic phase was dried over anhydrous Na_2SO_4 and concentrated under vacuum to give the crude (995 mg), which was purified by flash chromatography on silica gel (PE/EtOAc : 6/4 to 5/5) to give **15** as a yellow oil (396 mg, 63 % yield over two steps).

M.W.: ($C_{22}H_{29}NO_{10}$) 467.47 g/mol.
ESI-MS: *m/z* (%): 490.33 (100) $[M+Na]^+$, 506.33 (35) $[M+K]^+$, 956.58 (6) $[2M+Na]^+$, 972.00 (3) $[2M+K]^+$.
$[\alpha]_D^{26°C}$ = -42° (c = 0.873 g/100 mL, $CHCl_3$).
^{1}H NMR: (500 MHz, $CDCl_3$) δ: 7.39 – 7.35 (m, 4H, Ar), 7.34 – 7.28 (m, 1H, Ar), 5.28 – 5.22 (m, 2H, H-2 + H-3), 5.19 – 5.15 (m, 1H, NH), 5.11 (d, J = 3.4 Hz, 2H, CH_2-Ph), 5.06 (t, $J_{4\text{-}H}$ = 9.7 Hz, 1H, H-4), 4.73 (d, $J_{1\text{-}2}$ = 1.6 Hz, 1H, H-1), 3.83 (dq, $J_{5\text{-}4}$ = 9.8 Hz, $J_{5\text{-}6}$ = 6.3 Hz, 1H, H-5), 3.79 – 3.72 (m, 1H, 1H of H-a), 3.55 – 3.49 (m, 1H, 1H of H-a), 3.48 – 3.42 (m, 1H, 1H of H-b), 3.42 – 3.35 (m, 1H, 1H of H-b), 2.14 (s, 3H, Ac), 2.03 (s, 3H, Ac), 1.98 (s, 3H, Ac) 1.20 (d, $J_{6\text{-}5}$ = 6.4 Hz, 3H, H-6) ppm.
^{13}C NMR: (125 MHz, $CDCl_3$) δ: 170.3 (C_q, CO), 170.2 (C_q, CO), 170.1 (C_q, CO), 156.5 (C_q, CO), 136 (C_q, Ph), 128.7 (CH, Ph), 128.3 (CH, Ph), 97.7 (CH, C-1), 71.1 (CH, C-4), 69.8 (CH, C-3), 69.2 (CH, C-2), 67.4 (CH_2, C-a), 67.0 (CH_2, CH_2-Ph), 66.7 (CH, C-5) 40.8 (CH_2, C-b), 21.0 (CH_3, Ac), 20.9 (CH_3, Ac), 20.9 (CH_3, Ac), 17.5 (CH_3, C-6) ppm.

Synthesis of compound 16:

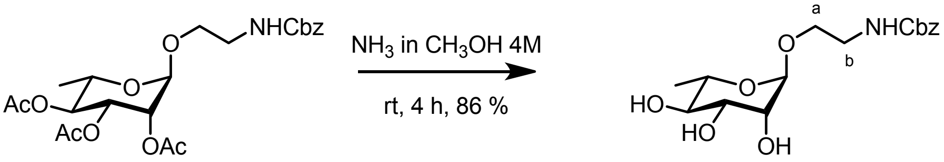

15 **16**

Compound **15** (393 mg, 0.85 mmol) was dissolved in 13 mL of a 4 M solution of NH_3 in CH_3OH. The mixture was stirred at room temperature for 4 hours after which the reaction mixture was evaporated to dryness and purified by flash chromatography on silica gel (CH_2Cl_2/CH_3OH : 9/1) to afford **16** (249 mg, 86 % yield) as a cerous solid.

M.W.: ($C_{16}H_{23}NO_7$) 341.36 g/mol.
ESI-MS: *m/z* (%): 364.33 (100) $[M+Na]^+$, 380.25 (39) $[M+K]^+$, 704.92 (29) $[2M+Na]^+$, 720.58 (4) $[2M+K]^+$.
$[\alpha]_D^{26°C}$ = -43° (c = 0.913 g/100 m in CH_3OH).
^{1}H NMR: (500 MHz, CD_3OD) δ: 7.37 – 7.33 (m, 4H, Ar), 7.32 – 7.26 (m, 1H, Ar), 5.08 (s, 2H, CH_2-Ph), 4.68 (d, $J_{1\text{-}2}$ = 1.8 Hz, 1H, H-1), 4.58 (s, 1H, NH), 3.81 (dd, $J_{2\text{-}3}$ = 3.5 Hz, $J_{1\text{-}2}$ = 1.8 Hz, 1H, H-2), 3.75 – 3.69 (m, 1H, one H of H-a), 3.65 (dd, $J_{3\text{-}4}$ = 9.5 Hz, $J_{3\text{-}2}$ = 3.5, 1H, H-3), 3.58 (dq, $J_{5\text{-}4}$ = 9.5 Hz, $J_{5\text{-}6}$ = 6.3 Hz, 1H, H-5), 3.52 – 3.44 (m, 1H, one H of H-a), 3.37 (at, $J_{4\text{-}H}$ = 9.4 Hz, 1H, H-4), 3.35 – 3.28 (m, 2H, H-b), 1.24 (d, $J_{6\text{-}5}$ = 6.3 Hz, 3H, H-6) ppm.
^{13}C NMR: (125 MHz, CD_3OD) δ: 158.9 (C_q, CO), 138.3 (C_q, Ph), 129.5 (CH, Ph), 129.0 (CH, Ph), 128.8 (CH, Ph), 101.7 (CH, C-1), 74.0 (CH, C-4), 72.3 (CH, C-2), 69.9 (CH, C-5), 67.5 (CH_2, CH_2-Ph), 67.3 (CH2, C-a), 41.7 (CH2, C-b), 18.0 (CH_3, C-6) ppm.

Synthesis of compound 17:

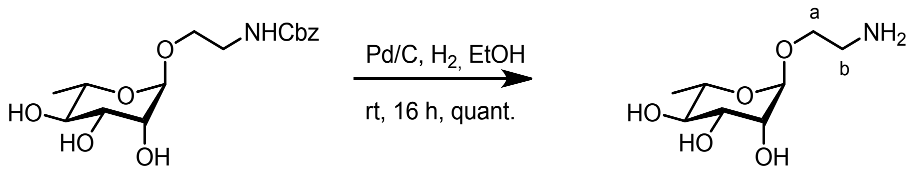

To a solution of **16** (249 mg, 0.73 mmol) in ethanol (10 mL), Pd/C (10 % wt, 189 mg) was added under a N_2 atmosphere. The reaction was stirred at room temperature, under H_2 atmosphere. After 16 h the suspension was filtered through a pad of Celite®, the solvent was removed under vacuum to give pure **17** (150 mg, quant. yield) as a cerous solid.

M.W.: ($C_8H_{17}NO_5$) 207.23 g/mol.
ESI-MS: *m/z* (%): 208.17 (100) $[M+H]^+$, 230.17 (23) $[M+Na]^+$.
$[\alpha]_D^{26°C}$ = -52° (c = 0.355 g/100 mL in CH_3OH).
^{1}H NMR: (500 MHz, CD_3OD) δ: 4.70 (d, $J_{1\text{-}2}$ = 1.8 Hz, 1H, H-1), 3.83 (dd, $J_{2\text{-}3}$ = 3.4 Hz, $J_{2\text{-}1}$ = 1.8 Hz, 1H, H-2), 3.72 (ddd, $J_{a\text{-}a'}$ = 10.4 Hz, $J_{a\text{-}b}$ = 6.1 Hz, $J_{a\text{-}b'}$ = 4.6 Hz, 1H, one H of H-a), 3.66 (dd J = 9.5, 3.4 Hz, 1H, H-3), 3.58 (dq, J = 9.5, 6.3 Hz, H-5), 3.45 (ddd, $J_{a\text{-}a'}$ = 10.4 Hz, $J_{a\text{-}b}$ = 6.4 Hz, $J_{a\text{-}b'}$ = 4.6 Hz, 1H, one H of H-a), 3.38 (at, $J_{4\text{-}H}$ = 9.5 Hz, 1H, H-4), 2.89 – 2.76 (m, 2H, H-b), 1.27 (d, $J_{6\text{-}5}$ = 6.3 Hz, 3H, H-6) ppm.
^{13}C NMR: (125 MHz, CD_3OD) δ: 101.9 (CH, C-1), 74.0 (CH, C-4), 72.4 (CH, C-3), 72.2 (CH, C-2), 69.9 (CH, C-5), 69.8 (CH_2, C-a), 42.1 (CH_2, C-b), 18.0 (CH_3, C-6) ppm.

Synthesis of compound 2:

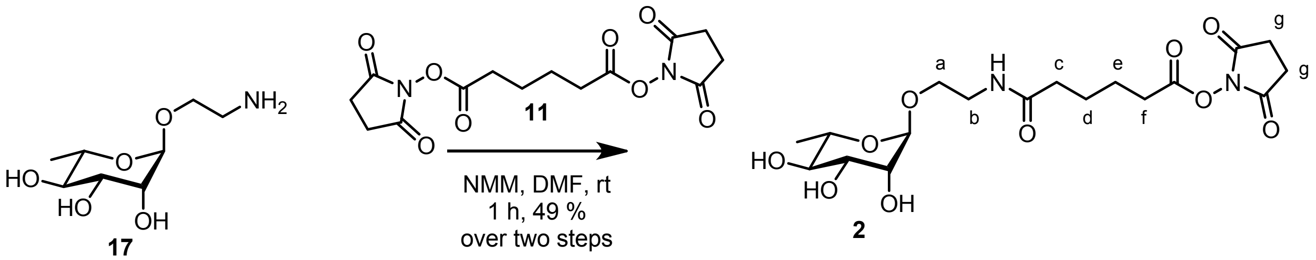

NMM (380 µL, 3.46 mmol) was added to a solution of **17** (150 mg, 0.72 mmol) in dry DMF (15 mL). After 20 minutes, **11** (695 mg, 2 mmol) was added. The mixture was stirred at room temperature for 1 hour after which the mixture was evaporated to dryness and purified via flash chromatography on silica gel ($CHCl_3/CH_3OH$: 80/20) to afford **2** (153 mg, 49 % yield over two steps) as a white foam.

M.W.: ($C_{18}H_{28}N_2O_{10}$) 432.43 g/mol.
ESI-MS: *m/z* (%): 455.42 (100) $[M+Na]^+$, 471.33 (8) $[M+K]^+$.
$[\alpha]_D^{26°C}$ = -31° (c = 0.605 g/100 mL in CH_3OH).
^{1}H NMR: (500 MHz, CD_3OD) δ: 4.68 (d, $J_{1\text{-}2}$ = 1.7 Hz, 1H, H-1), 3.81 (dd, $J_{2\text{-}3}$ = 3.5 Hz, $J_{2\text{-}1}$ = 1.7 Hz, 1H, H-2), 3.72 (ddd, $J_{a\text{-}a'}$ = 10.2 Hz, $J_{a\text{-}b}$ = 6.5 Hz, $J_{a\text{-}b'}$ = 4.7 Hz, 1H, one H of H-a), 3.69 – 3.62 (m, 1H, H-3),

3.56 (dq, $J_{5\text{-}4}$ = 9.4 Hz, $J_{5\text{-}6}$ = 6.2 Hz, 1H, H-5), 3.45 (ddd, $J_{a\text{-}a'}$ = 10.2 Hz, $J_{a\text{-}b}$ = 6.5 Hz, $J_{a\text{-}b'}$ = 4.6 Hz, 1H, one H of H-a), 3.44 – 3.33 (m, 3H, H-4 + H-b), 2.83 (s, 4H, H-g), 2.69 – 2.65 (m, 2H, H-c), 2.28 – 2.21 (m, 2H, H-f), 1.79 – 1.70 (m, 3H, H-d + H-e), 1.25 (d, $J_{6\text{-}5}$ = 6.3 Hz, 3H, H-6) ppm.
^{13}C NMR: (125 MHz, CD_3OD) δ: 175.7 (C_q, CO), 171.9 (C_q, CO), 170.1 (C_q, CO), 101.6 (CH, C-1), 73.9 (CH, C-4), 72.3 (CH, C-3), 72.1 (CH, C-2), 69.8 (CH, C-5), 66.9 (CH_2, C-a), 36.4 (CH_2, C-c), 31.2 (CH_2, C-f), 26.5 (CH_2, C-g), 26.0 (CH_2, C-d), 25.1 (CH_2, C-e), 18.0 (CH_3, C-6) ppm.

Material and Methods

Cell culture

The MDA-MB-231 is a highly aggressive, invasive, and poorly differentiated triple-negative breast cancer (TNBC) cell line as it lacks estrogen receptor (ER), and progesterone receptor (PR) expression, as well as human epidermal growth factor receptor 2 (HER2). MDA-MB-231 cells were cultivated in DMEM medium complete with inactivated 10% fetal bovine serum (FBS), 1% penicillin/streptomycin and 1% L-glutamine.
MCF-7 represents an ER-positive breast cancer model expressing both ERα and Erβ, PR as well as HER2 receptors. MCF-7 cells were cultivated in EMEM medium supplemented with 10% FBS, 1% penicillin/streptomycin, 1% L-glutamine, 1% non-essential amino acids, 1% sodium pyruvate and 1% insulin. Both cell cultures were kept in an incubator at 37°C in a humidified atmosphere with a CO_2 pressure of 5%.
Human Peripheral Blood Mononuclear Cells (PBMC), were isolated from the buffy coat of healthy volunteers after their informed consent. PBMC were separated from whole blood by density gradient (ficoll) centrifugation. Isolated PBMC were cultivated in RPMI medium, completed with 10% FBS, 1% Kanamycin solution, 1% L-glutamine, 1% non-essential amino acids and 1% sodium pyruvate.
To evaluate the expression of PD-L1 on cell lines, MDA MB 231 and MCF-7 cells were harvested labelled with PE-conjugated anti-PD-L1 antibody and the level of PD-L1 expression analyzed by flow cytometry.

Cell co-culture

For co-culture experiments, each cell line was plated in a 96 well tissue culture-treated plate at a concentration of 30000 cells/well. Cells were kept in a humidified incubator at 37°C in 5% CO_2 for 24 h to allow the cell to adhere. PBMC were activated or not activated by PHA 10 μg/mL overnight treatment.
To establish co-culture cell lines were treated with 10 μg/mL or 50 μg/mL of WT PD-1, HACTR-PD-1, **1**- or **2**-HACTR-PD-1-L-rhamnose for 1 hour, followed by the addition of inactivated/activated PBMCs (3 x 10^5 cells/well). To distinguish the effects of co-culturing PBMCs with breast cancer cells from PBMC activation, two controls were used; activated PBMCs cultured alone (negative control) or co-cultured with breast cancer cells from each cell line, in the absence of recombinant or natural proteins.

Proliferation assay

To evaluate the effect of WT PD-1, HACTR-PD-1 or HACTR-PD-1-L-rhamnose on T cell proliferation PBMC were labelled with 0.25 mM carboxyfluorescein succinimidyl ester (CFSE) in serum-free PBS for 30 min at 37°C. FBS was then added to stop the reaction, and cells were washed several times with completed RPMI-1640. CFSE-labelled PBMC were added to pleated MDA-MB-231 or MCF-7 (untreated/treated 1h with WT PD-1, HACTR-PD-1, **1**- or **2**-HACTR-PD-1-L-rhamnose) and stimulated with 10 μg/mL of PHA. After 6 days of co-culture, PBMC were harvested, and T cell proliferation analyzed by FACS.

Cytotoxicity assay

The effect of WT PD-1, HACTR-PD-1 or HACTR-PD-1-L-rhamnose on T cell cytotoxic activity was analyzed using Calcein-AM cytotoxicity assay. MDA-MB-231 and MCF-7 cells were labelled with 1 mM CAM at 37°C for 15 min, washed, and seeded in a 96-well plate at a density of 3 x 10^4 cells in 50 μL per well. The following day, labelled target cells were treated with 10 μg/mL or 50 μg/mL of WT PD-1, HACTR-PD-1, **1**- or **2**-HACTR-PD-1 for 1 hour and incubated at a 1:10 ratio with overnight stimulated PBMC (PBMC stimulation

was performed as described in paragraph Cell Co-Culture). Each plate included target cells alone, as controls, for spontaneous cell death measurements. Plates were incubated at 37°C in a humidified atmosphere with 5% CO_2 for 24h. After incubation, the cells of each well were harvested, washed and labelled with propidium iodide (PI), and the cytotoxicity was measured by flow cytometry (FACS). Live target cells were identified as CAM^{high}/PI^{-} population, whereas killed target cells were CAM^{low}/PI^{+} and the effector cells were CAM^{-} (at least 10-fold less fluorescent than killed target cells). After gating on target cells, cytotoxicity was calculated as the % increase in CAM^{low}/PI^{+} population relative to target cells alone [cytotoxicity, % = (CAM^{low}/PI^{+} in experimental wells – CAM^{low}/PI^{+} in control wells)/CAM^{high}/PI^{-} in control well x 100]. The mean cytotoxicity % SEM for each condition was calculated from three replicate experimental wells.

THP1 cell culture and differentiation

THP1 cell line was obtained from ATCC and maintained in RPMI 1640 medium supplemented with 10% of fetal bovine serum (FBS), 2 mmol/L L-glutamine (Immunological Sciences, Rome, Italy) and 1 mg/mL kanamycin (Sigma-Aldrich Milan, Italy). THP1 cells (2 x 10^5 cells/mL) were seeded in 6 multiwell and differentiated into macrophages (M0) by 24h incubation with 150 nM phorbol 12-mystrate-13-acetate (PMA) (Sigma-Aldrich) followed by 24h incubation in RMPI medium. Macrophages were polarized into M1 macrophages by incubation with 0.5 mg/mL lipopolysaccharide (LPS) (Sigma Aldrich, Milan Italy).

Flow cytometry

Flow cytometry analyzes were performed using the Accuri C6 (Thermo Fisher Scientific, Italy). Forward (FCS) and side (SSC) scatters were used to identify cell populations and measure size and granularity of the cells. Auto-fluorescence was recorded by analyzing unstained cells in the FL-1 channel (blue laser; excitation 488, emission 530/30). For detection of cell surface markers 1 mg/mL of monoclonal mouse anti-human antibodies CD14-FITC, CD86-PE, CD11b- PeCy7 were used for each sample. THP1 cells (2 x 10^5 cells/mL) were seeded in 6 MW and differentiated into macrophages as described. Differentiate macrophages (M0) were treated with increasing concentrations (0.1 – 10 mg/mL) of tested compounds or with 0.5 mg/mL (LPS). After 24h incubation, cells were harvested, washed, incubated with the antibodies for 30 minutes on ice in the dark. Labelled samples were washed, resuspended in PBS and analyzed by FACS, for each sample 10000 events were recorded. All data was analyzed using FCS express 7 (Flow cytometry software, DeNovo software).

ELISA assay

To evaluate the effect of new PD-1 mutants on T helper/reg cell activity co-culture experiments were performed as described previously. After 48h of co-culture, plates were centrifuged at 1500 rpm for 5 minutes and cell supernatants collected and stored at -80°C until the analysis. IFN-γ and IL-10 quantification in the culture media was performed by ELISA, following the manufacturer's instructions. Absorbance at 450 nm was monitored with a microplate reader. THP1 cells (2 x 10^5 cells/mL) were seeded in 6 MW and differentiated into macrophages as described. Differentiate macrophages (M0) were treated with increasing concentrations (0.1 – 10 mg/mL) of tested compounds or with 0.5 mg/mL (LPS). After 24h incubation culture media were collected and stored in –80°C until analysis. Levels of IL-8, TNF-a and IL-10 were measured by ELISA assay according to manufacturer's guidelines (Biolegend® San Diego, CA, USA).

Cell subset characterization

To evaluate the effect of WT PD-1, HACTR-PD-1, **1**- or **2**-HACTR-PD-1 on T cell subset frequency co-culture experiments were performed as described previously. After 72 h of co-culture PBMC were harvested labelled for 30 min on ice with anti-human CD3, CD4, CD8 and CD25 and washed 2 times with PBS. CD3, CD4, CD8, and CD25 frequency were analyzed by FACS.

Statistical analysis

Results are expressed as means ± SEM of at least three independent experiments. Independent experiments were conducted using PBMC from at least 3 different donors. Statistical significance was evaluated by the one-way ANOVA followed by the Student's t test for unpaired populations, using Graph Pad Prism 9 (Graph Pad Software, Inc., San Diego, CA, USA). Differences were considered statistically significant when $p < 0.05$.

Expression and purification of human wild-type PD-L1

Escherichia coli BL21 (DE3) cells were transformed with pET-21a (+) plasmid encoding PD-L1 gene. In order to obtain uniformly isotopically enriched PD-L1 [U-^{15}N], the cells were cultured in M9 Minimal Medium supplied with 1.1 g ^{15}N- NH_4Cl, 1 mL of 0.1 mg/mL solution of ampicillin, 1 mL of 1 mg/mL of thiamine, 1 mL of 1 mg/mL of biotin, 1 mmol·dm^{-3} $MgSO_4$, 0.3 mmol·dm^{-3} $CaCl_2$; they were allowed to grown at 37 °C until OD_{600} reached 0.8 and then overexpression was induced with 1 mmol·dm^{-3} isopropyl β-D-1-thiogalactopyranoside. The cultures were further incubated in agitation at 37 °C, overnight, and then harvested by centrifugation at 6500 rpm (JA-10 Beckman Coulter) for 15 min at 4°C. In all instances the pellet was suspended, at first, in 50 mmol·dm^{-3} Tris-HCl pH 8.0, 200 mmol·dm^{-3} NaCl, 10 mmol·dm^{-3} β-mercaptoethanol, 10 mmol·dm^{-3} EDTA, (50 mL per litre of culture) and sonicated for 30 seconds 10 times on ice at 4 °C. The suspension was centrifuged at 40,000 rpm (F15-6x100y Thermo Scientific) for 40 min and the supernatant was discarded. The recovered pellet was resuspended in 50 mmol·dm^{-3} Tris-HCl pH 8.0, 200 mmol·dm^{-3} NaCl, 10 mmol·dm^{-3} β-mercaptoethanol, 6 mol·dm^{-3} Guanidinium Chloride (25 mL per litre of culture) and newly incubated at 4 °C overnight under magnetic stirring. Again, the suspension was centrifuged at 40,000 rpm (F15-6x100y Thermo Scientific) for 40 min. The pellet was discarded, whereas the supernatant containing the denatured protein solution was diluted in a refolding buffer containing 0.1 mol·dm^{-3} Tris–HCl, pH 8.5, 1 mol·dm^{-3} arginine, 0.25 mmol·dm^{-3} reduced glutathione and 0.25 mmol·dm^{-3} oxidised glutathione. The solution was incubated at 4 °C under stirring, for 12-18 h, clarified by passing a 0.45-µm filter and then dialyzed extensively against 10 mmol·dm^{-3} Tris, pH 8.0, 20 mmol·dm^{-3} NaCl. The protein solution was concentrated with an Amicon® Stirred Cell and then purified by size exclusion chromatography using a HiLoad Superdex 26/60 75pg (GE Healthcare) column previously equilibrated in 10 mmol·dm^{-3} Tris-HCl pH 8.0 and 20 mmol·dm^{-3} NaCl.

NMR measurements

Solution NMR experiments for backbone resonance assignment [3D HNCA, HNCACB, CBCA(CO)NH, HNCO, HN(CA)CO][1–3] were performed on [U-^{13}C, ^{15}N] samples of the HACTR-PD-1 mutant (at the concentration of 350 µmol·dm^{-3}) in the same water buffer solution used for PD-L1 resonance assignment[4] [10 mmol·dm^{-3} Tris, pH 8, 20 mmol·dm^{-3} NaCl]. For 3D HNCACB non-uniform random sampling at 62% and compressed-sensing reconstruction was used.[5] A 3D HNCA was also recorded at a lower pH [buffer: 20 mmol·dm^{-3} HEPES, pH 6.8, 20 mmol·dm^{-3} NaCl, 0.1% NaN_3] to identify a higher number of spin systems. All solution spectra were recorded at 298 K on Bruker AVANCE III and AVANCE NEO NMR spectrometers, operating at 950 and 500 MHz, ^{1}H Larmor frequency, (22.3 T and 11.7 T), respectively, equipped with triple resonance cryo-probes. Secondary structure prediction was performed with TALOS+[6] by using the chemical shifts of HN, N, C', Cα, and Cβ as input data.

NMR Titrations of the functionalized HACTR-PD-1 mutant with PD-L1

The interactions of the free and functionalized HACTR-PD-1 mutant with PD-L1 have been investigated through solution NMR titrations. During the NMR titration, increasing aliquot of PD-L1 [to reach the concentrations of 12.5, 25, 50 µmol·dm^{-3} in solution] were added to the solution of free [U-^{15}N] HACTR-PD-1 mutant or [U-^{15}N] HACTR-PD-1 mutant functionalized with PEG1000, PEG5000 and rhamnose-derivative [at protein concentration of 50 µmol·dm^{-3} in 10 mmol·dm^{-3} Tris, pH 8, 20 mmol·dm^{-3} NaCl]. 2D ^{1}H-^{15}N HSQC NMR spectra were recorded after each addition on Bruker AVANCE III and AVANCE NEO NMR spectrometers, operating at 950 and 900 or 700 MHz, ^{1}H Larmor frequency, (22.3 T, 21.1 T and 16.4 T) respectively, equipped with triple resonance cryo-probes. An NMR titration of [U-^{15}N] PD-L1 [50 µmol·dm^{-3}

in 10 mmol·dm^{-3} Tris, pH 8, 20 mmol·dm^{-3} NaCl] with free HACTR-PD-1 mutant was also performed on Bruker AVANCE III spectrometer, operating at 950 MHz, ^{1}H Larmor frequency (22.3 T); equivalent aliquots of HACTR-PD-1 [to reach the concentrations of 12.5, 25, 50 μmol·dm^{-3} in solution] were added to [U-^{15}N] PD-L1 solution.

HADDOCK calculations

The PDB structure of the complex between the HAC-PD-1 mutant and PD-L1 (PDB code: 5IUS)[7] was used as a starting input to generate the structures of the different mutants (K131T/K135R, K131R/K135R, K131Q/K135Q, K131T/K135Q) with PyMOL. The residue K135 is at the interface between the two proteins in the complex and faces an aspartate (D61) on the PD-L1 side. For this reason, in most of the complexes we kept the positive charge in this position by mutating the lysine to an arginine. Then, the obtained complexes were minimized through MODELLER[8] and finally water refined using the HADDOCK web-portal on the WeNMR GRID (http://www.wenmr.eu), using the refinement interface.[9,10] Reference calculations were also performed for the 5IUS and 4ZQK[11] PDB structures, which were refined and scored in the same way. The interacting energy of the complexes was also analyzed on the PRODIGY server[12,13] after MODELLER and HADDOCK minimization. The model of the HACTR-PD-1/PD-L1 complex was obtained by performing docking calculations with the software HADDOCK 2.4 on the WeNMR GRID (http://www.wenmr.eu).[9,10] In all calculations, during the rigid-body docking, 1000 complexes were generated, then 200 structures were selected for the semi-flexible simulated annealing in torsion angle space, and finally refined in Cartesian space with explicit solvent. The model structure of HACTR-PD-1 and PD-L1, obtained with the previous calculations, provided the input coordinates for the proteins. The NMR titrations of [U-^{15}N] HACTR-PD-1 with PD-L1 and [U-^{15}N] PD-L1 with HACTR-PD-1, analyzed with the PICASSO web server,[14] provided experimental ambiguous restraints ("active residues") to drive the docking calculation.

ITC Titration of the HACTR-PD-1 mutant with PD-L1

Isothermal titration microcalorimetry experiments were performed at 298 K with a VP-ITC microcalorimeter (MicroCal, Inc., Northampton, MA). After an initial injection of 1 μL, aliquots of 13 μL of 300 μmol·dm^{-3} HACTR-PD-1 were stepwise injected into the sample cell containing a 30 μmol·dm^{-3} solution of PD-L1 until complete saturation was obtained. All experiments were performed in 10 mmol·dm^{-3} Tris-HCl at pH 8.0 with 20 mmol·dm^{-3} NaCl. Heats of dilution were measured by injecting the ligand solution into the buffer, and the obtained values were subtracted from the binding heats. The thermodynamic parameters and K_A values were calculated by fitting the data to a single binding site model with the Origin 7.0 software (MicroCal, Inc.).

Table S1. HADDOCK-scores evaluated on the best four structures of the complexes for the mutants considered in the study.

Complex	HADDOCK-score
4ZQK	-112 ± 2
5IUS	-119 ± 3
5IUS RR	-114 ± 2
5IUS TR	-113 ± 1
5IUS TQ	-108 ± 2
5IUS QQ	-96 ± 2

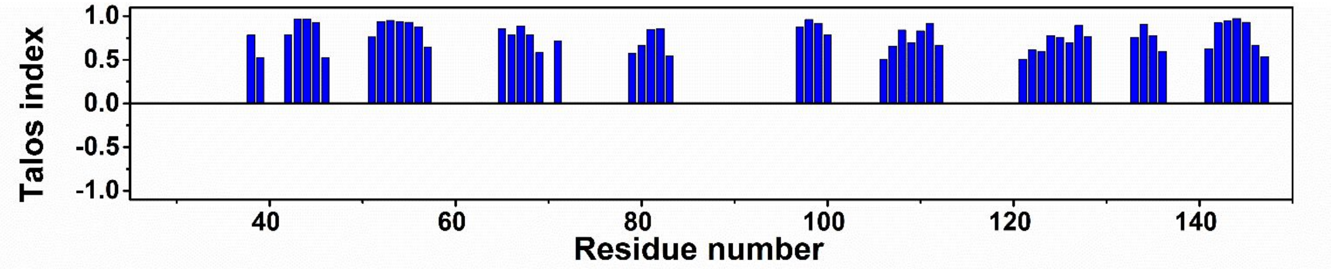

Figure S1. Secondary structure prediction obtained by the program TALOS+ using the experimental values of chemical shifts of HN, N, C’, Cα, and Cβ atoms as input data. The blue bars indicate the β-strand propensity.

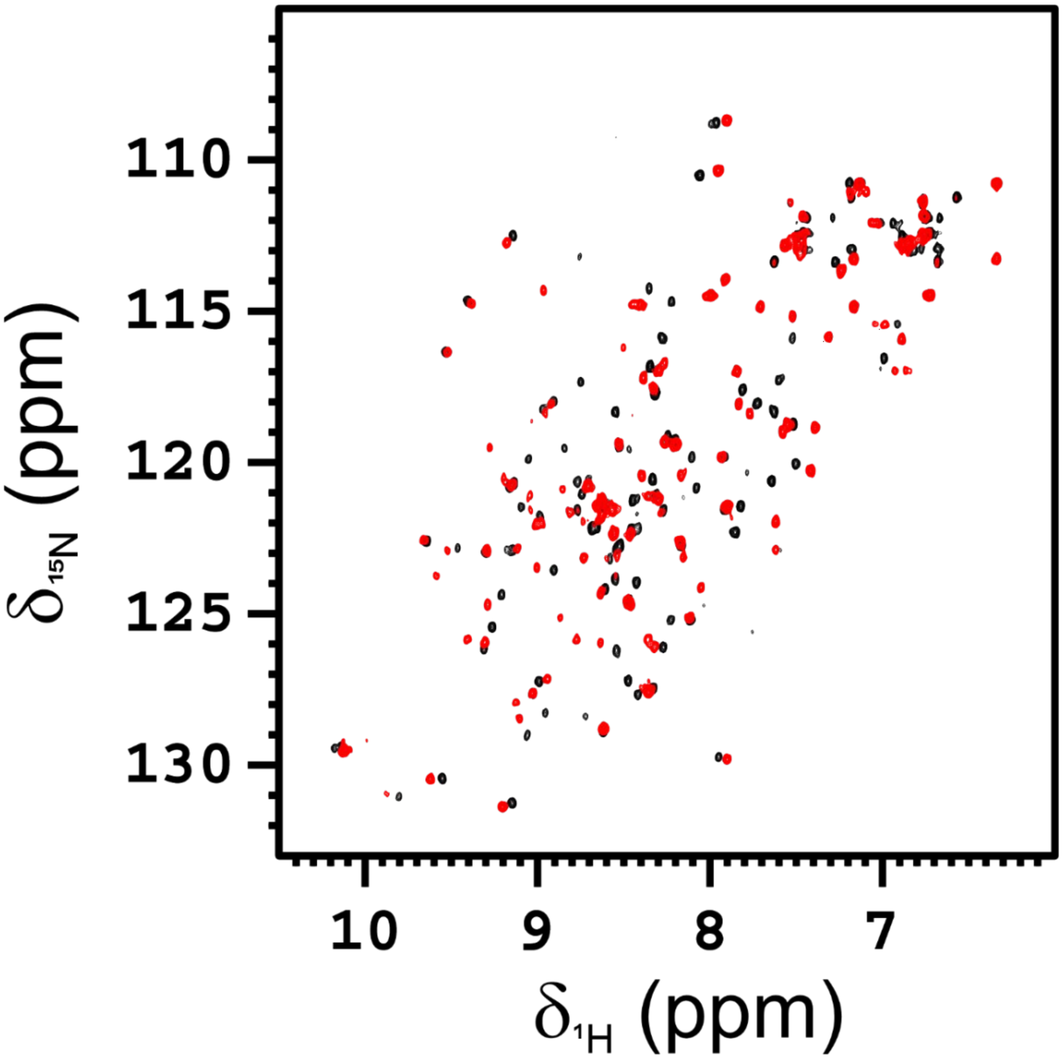

Figure S2. 2D ^{1}H-^{15}N HSQC overlaid spectra of free HACTR-PD-1 (black) with respect to HACTR-PD-1 in the presence of PD-L1 (in 1:1 molar ratio, red). The spectra were acquired on a spectrometer operating at 900 MHz, ^{1}H Larmor frequency, and 298 K. The spectrum of the complex was acquired with a higher number of scans than the reference spectrum.

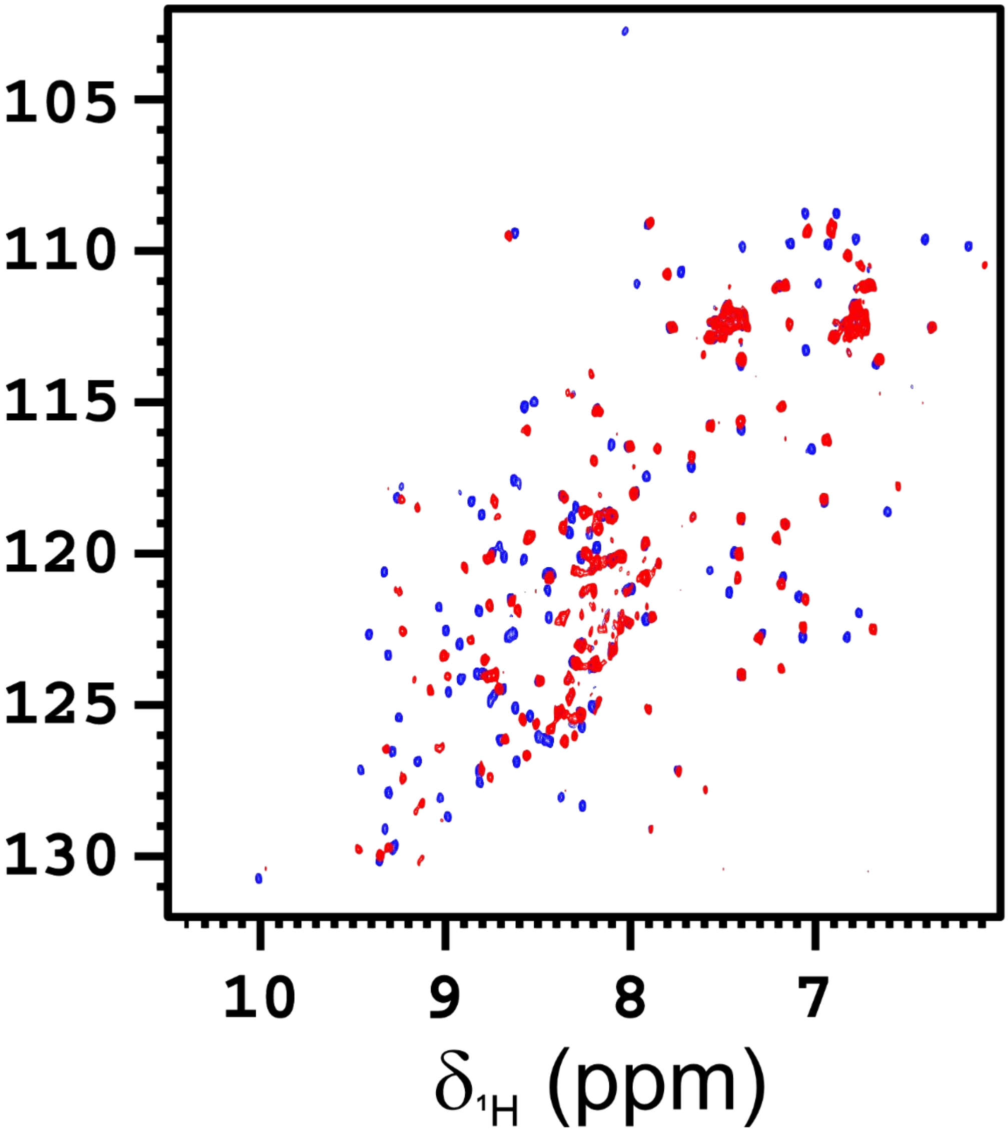

Figure S3. 2D ^{1}H-^{15}N HSQC overlaid spectra of free PD-L1 (blue) and PD-L1 in the presence of HACTR-PD-1 (in 1:1 molar ratio, red. The spectra were acquired on a spectrometer operating at 900 MHz, ^{1}H Larmor frequency, and 298 K. The spectrum of the complex was acquired with a higher number of scans than the reference spectrum.

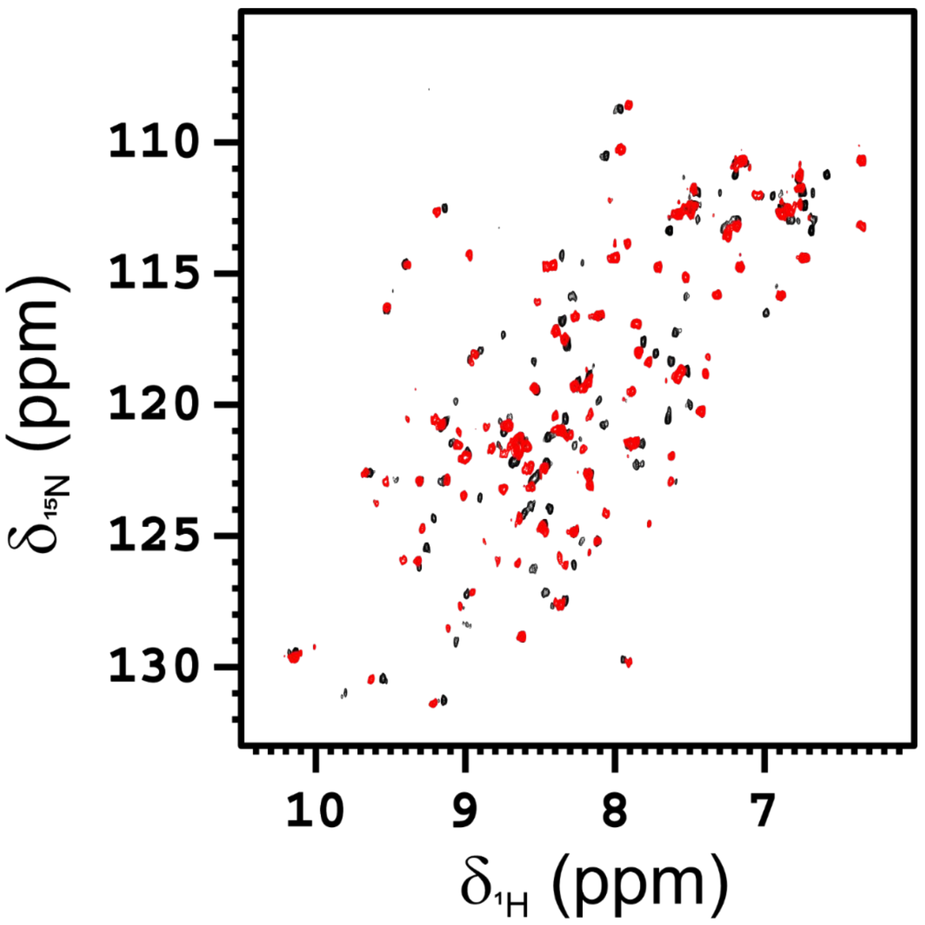

Figure S4. 2D ^{1}H-^{15}N HSQC overlaid spectra of HACTR-PD-1 conjugated with PEG 5 kDa (black) and HACTR-PD-1 conjugated with PEG 5 kDa in the presence of PD-L1 (in 1:1 molar ratio, red). The spectra were acquired on a spectrometer operating at 950 MHz, ^{1}H Larmor frequency, and 298 K. The spectrum of the complex was acquired with a higher number of scans than the reference spectrum.

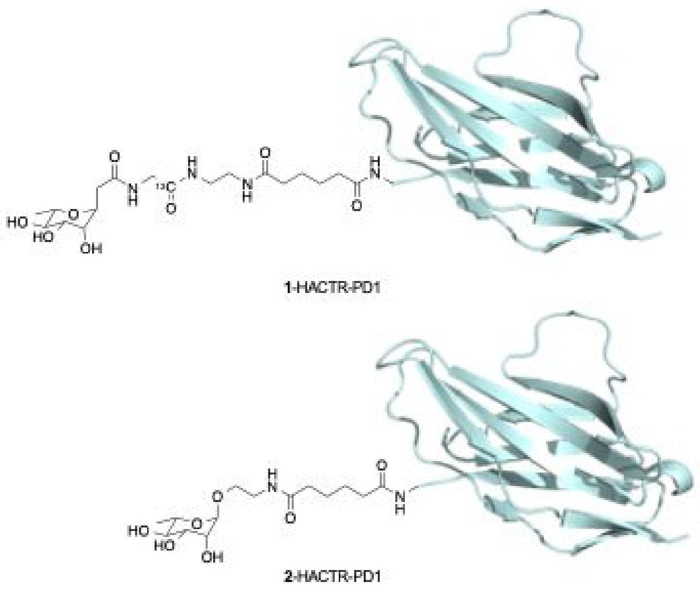

Figure S5. Rhamnosylated mutants **1**- HACTR-PD-1 and **2**- HACTR-PD-1.

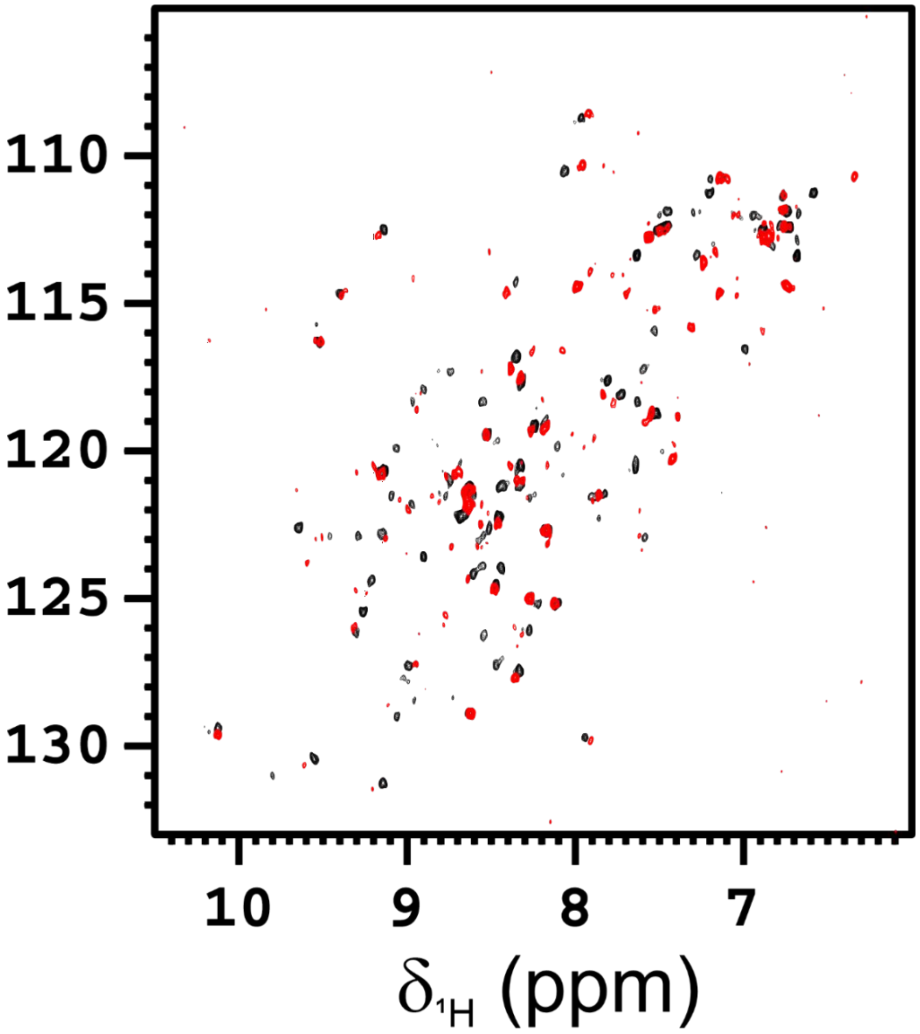

Figure S6. 2D ^{1}H-^{15}N HSQC overlaid spectra of HACTR-PD-1 conjugated with L-rhamnose, **1**- HACTR-PD-1 (black) and HACTR-PD-1 conjugated with L-rhamnose in the presence of PD-L1 (in 1:1 molar ratio, red). The spectra were acquired on a spectrometer operating at 700 MHz, ^{1}H Larmor frequency, and 298 K. The spectrum of the complex was acquired with a higher number of scans than the reference spectrum.

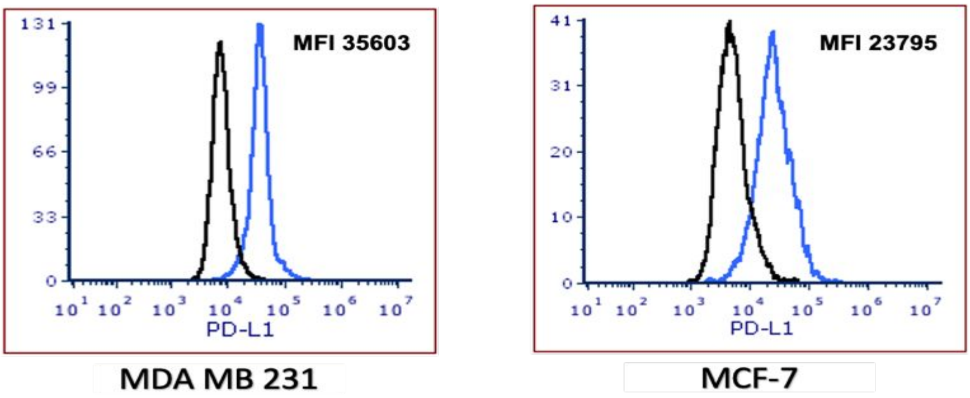

Figure S7. PD-L1 level expression on MDA MB 231 and MCF-7 breast cancer cell lines. Representative overlapping histogram plots of PD-L1 level of expression on MDA MB 231 and MCF-7 cells analyzed by FACS; isotype control black; PD-L1 1 blue. Data represent one of at least three independent experiments.

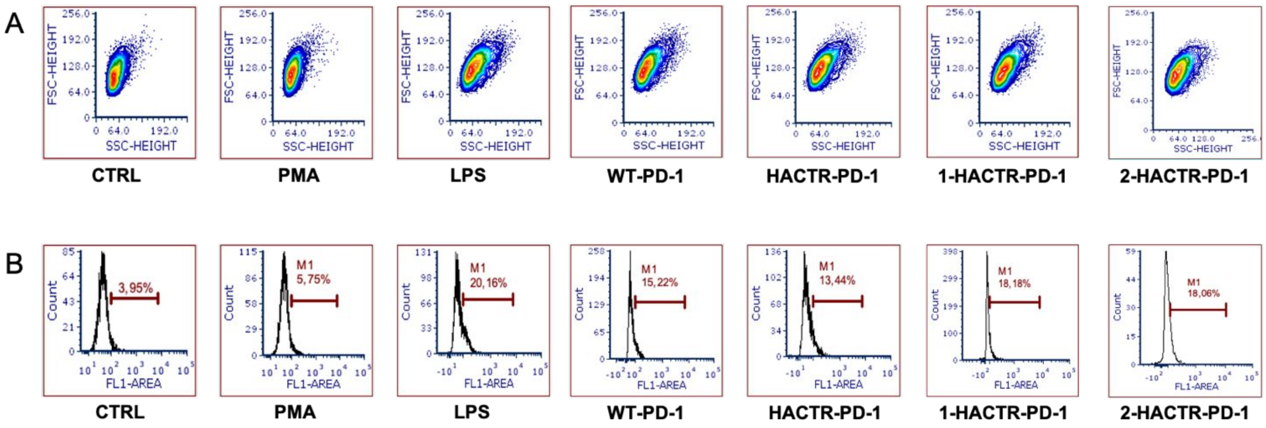

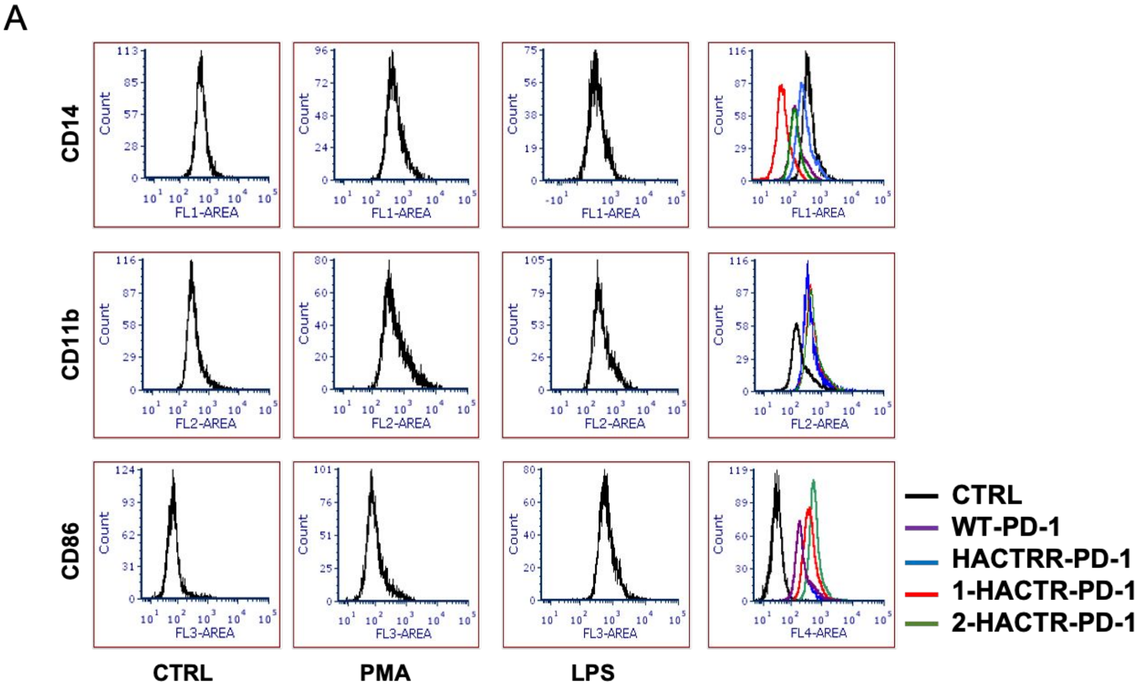

Figure S8. Effects of test compounds on THP1 cells morphological changes. (A) Representative forward light scatter and side light scatter plots of THP1 cells treated 24 h with 150 nM of PMA and of M0 treated (24 h) with 0.5 µg/mL of LPS or 1 µg/mL of tested compounds. (B) Representative histograms of autofluorescence of THP1 cells treated 24h with 150 nM of PMA and of M0 treated (24 h) with 0.5 µg/mL of LPS or 1 µg/mL of tested compounds. Data are representative of at least three experiments.

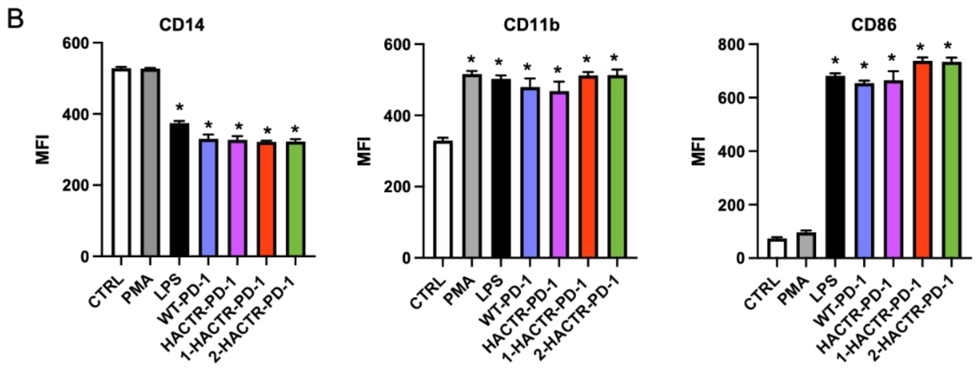

Figure S9. Effects of compounds on marker surface expression on differentiated THP1 cells. (A) Representative flow cytometry histograms showing CD14, CD11b, and CD86 expression on M0 treated (24h) with WT-PD-1, HACTR-PD-1, **1**-HACTR-PD-1-L-rhamnose or **2**-HACTR-PD-1-L-rhamnose. Level of expression of CD14 (B), CD11b (C), and CD86 (D) on M0 treated (24 h) with WT-PD-1, HACTR-PD-1, **1**-HACTR-PD-1-L-rhamnose or **2**-HACTR-PD-1-L-rhamnose. Results represent mean ± SEM of at least three independent experiments. * ≤0.05 treated *vs.* control (CTRL).

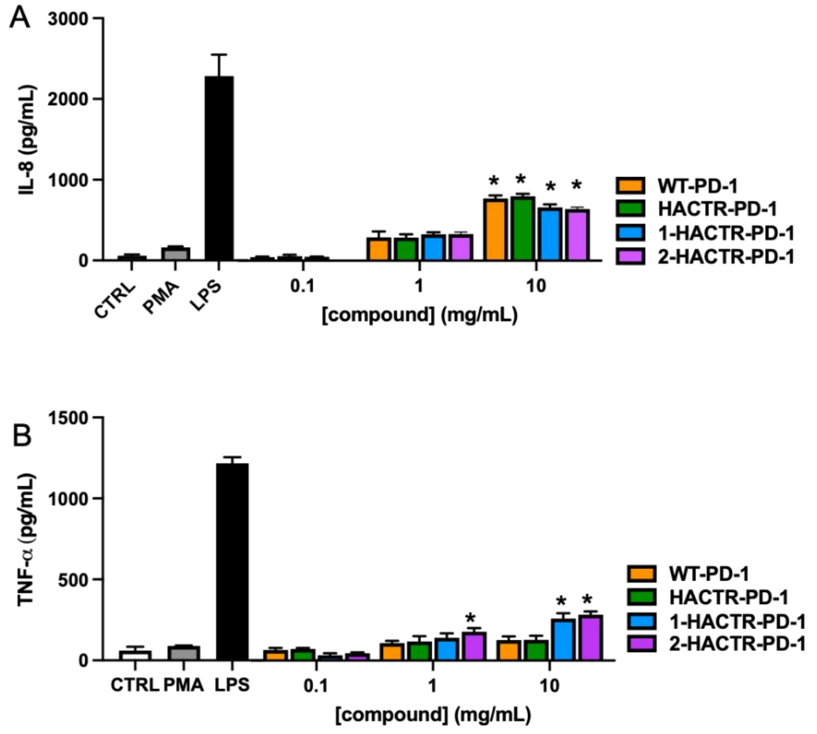

Figure S10. Effects of tested compound on cytokines secretion in differentiated THP1 cells. PMA differentiated THP1 cells were treated with 0.5 µg/mL of LPS or 1 µg/mL of tested compounds for 48h, cells culture medium harvested and IL-8 (A) and TNF-α (B) levels measured by ELISA assay. Results represent mean ± SEM of at least three independent experiments. * ≤0.05 treated *vs.* control (CTRL).

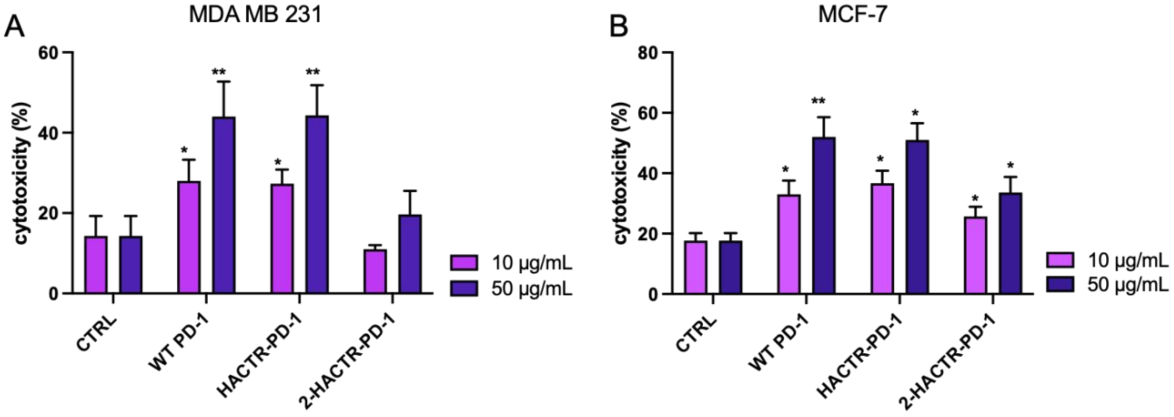

Figure S11. Effects of WT PD-1, HACTR-PD-1 and **2**-HACTR-PD-1 on T cell-mediated breast cancer cell cytotoxicity. CAM labelled cancer cells were treated with 10 or 50 μg/mL of PD-1, HACTR-PD-1 or **2**-HACTR-PD-1 for 1h and then incubated with PHA-stimulated PBMC. After 24h of co-culture breast cancer cells were harvested and the level intensity of CAM was analyzed by FACS. A. PBMC mediated cytotoxicity against MDA MB 231 cell line in presence/absence of WT PD-1, HACTR-PD-1 or **2**-HACTR-PD-1; B. PBMC mediated cytotoxicity against MCF-7 cell line in presence/absence of WT PD-1, HACTR-PD-1 or **2**-HACTR-PD-1. Results are expressed as the mean ± SEM of at least three independent experiments run in triplicate using PBMC from three different donors. *$p \leq 0.05$ natural/recombinant protein treated vs untreated co-cultures; **$p \leq 0.01$ natural/recombinant protein treated vs untreated co-cultures.

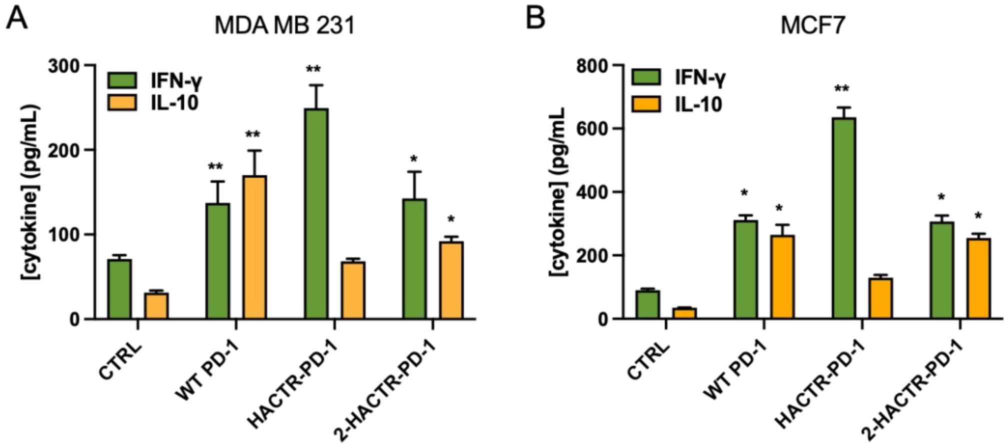

Figure S12. Effects of WT PD-1, HACTR-PD-1 and **2**-HACTR-PD-1 on T helper or Treg cytokine release. Cancer cells were treated with 10 μg/mL of WT PD-1, HACTR-PD-1 or **2**-HACTR-PD-1 for 1h and then incubated with PHA-stimulated PBMC. After 48h of cell culture media were collected and IFN- and IL-10 measured by ELISA. A. IFN- and IL-10 released by PBMC co-cultured with MDA MB 231 cell line in presence/absence of WT PD-1, HACTR-PD-1 or **2**-HACTR-PD-1; B. IFN-γ and IL-10 released by PBMC co-cultured with MCF-7 cell line in presence/absence of WT PD-1, HACTR-PD-1 or **2**-HACTR-PD-1. Results are expressed as the mean ± SEM of at least three independent experiments run in triplicate using PBMC from

different donors. * p≤0.05 natural/recombinant protein treated vs untreated co-cultures; **p≤0.01 natural/recombinant protein treated vs untreated co-cultures.

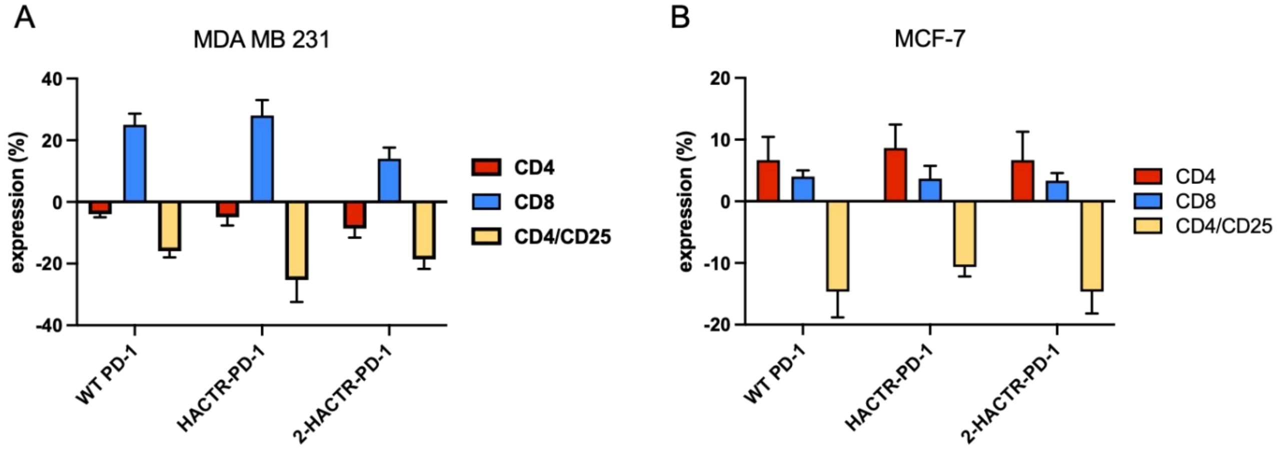

Figure S13. Effects of WT PD-1, HACTR-PD-1 and **2**-HACTR-PD-1 on T subset percentage. Cancer cells were treated with 10 μg/mL of PD-1, HACTR-PD-1 or **2**-HACTR-PD-1 for 1h and then incubated with PHA-stimulated PBMC. After 72h of co-culture, PBMC were harvested, labelled with CD3, CD4, CD8, and CD25 monoclonal antibodies and the percentage of CD4, CD8 and CD4-CD25 positive cells evaluated by FACS. A. CD4, CD8 and CD4CD25 percentage after co-cultured with MDA MB 231 cell line in presence/absence of WT PD-1, HACTR-PD-1 or **2**-HACTR-PD-1; B. CD4, CD8 and CD4CD25 after co-cultured with MCF-7 cell line in presence/absence of WT PD-1, HACTR-PD-1 or **2**-HACTR-PD-1. Results are expressed as mean ± SEM of at least three independent experiments run in triplicate using PBMC from different donors.

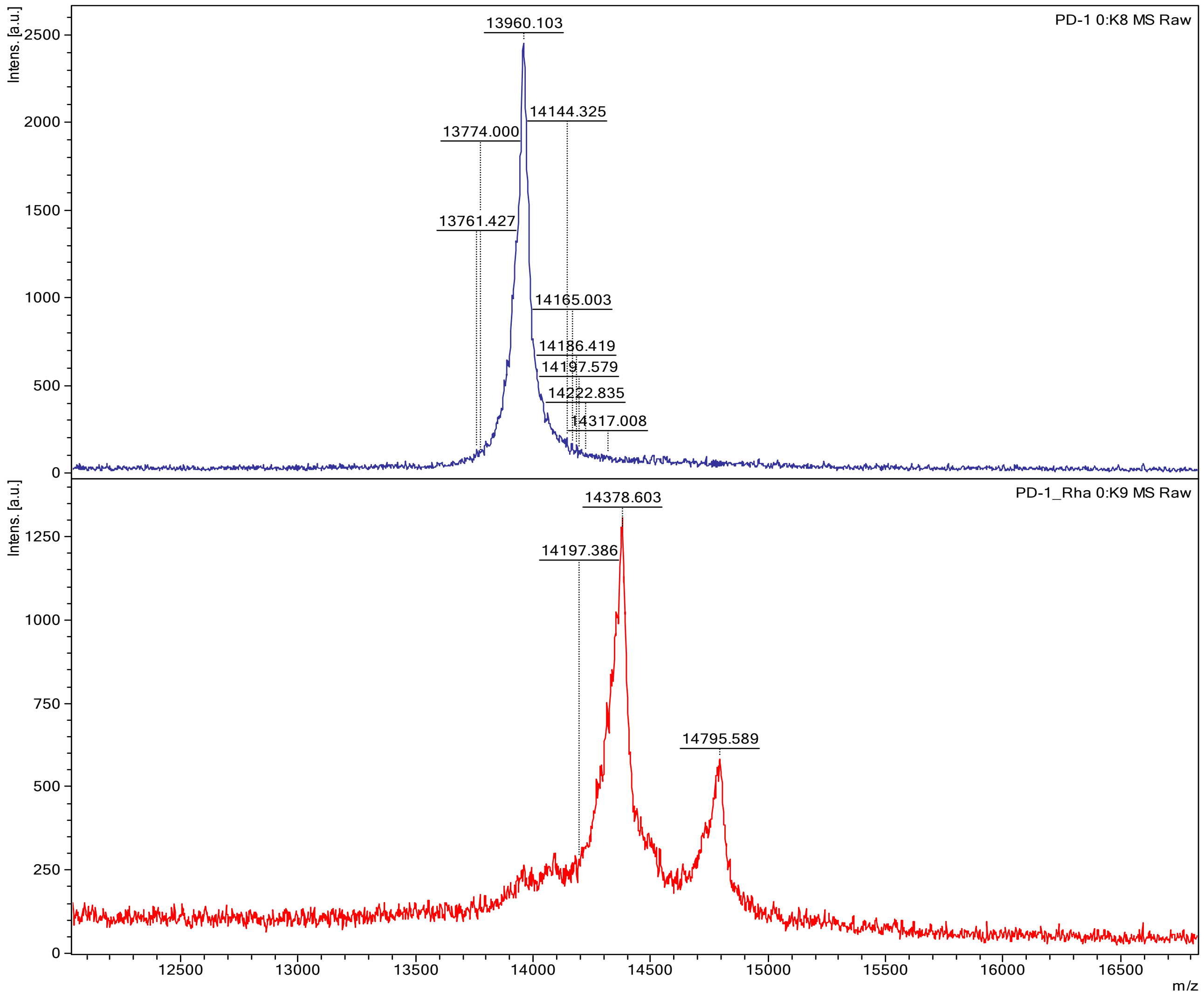

Figure S14. Detail of the MALDI-MS spectra of HACTR-PD-1 and **1**-HACTR-PD-1 collected on a Bruker Daltonics Ultraflex III TOF/TOF.

References

(1) Grzesiek, S.; Bax, A. Improved 3D Triple-Resonance NMR Techniques Applied to a 31 KDa Protein. *Journal of Magnetic Resonance (1969)* **1992**, *96* (2), 432–440. https://doi.org/10.1016/0022-2364(92)90099-S.

(2) Schleucher, J.; Sattler, M.; Griesinger, C. Coherence Selection by Gradients without Signal Attenuation: Application to the Three-Dimensional HNCO Experiment. *Angewandte Chemie International Edition in English* **1993**, *32* (10), 1489–1491. https://doi.org/10.1002/anie.199314891.

(3) Kay, L. E.; Xu, G. Y.; Yamazaki, T. Enhanced-Sensitivity Triple-Resonance Spectroscopy with Minimal H2O Saturation. *Journal of Magnetic Resonance, Series A* **1994**, *109* (1), 129–133. https://doi.org/10.1006/jmra.1994.1145.

(4) Rizzo, D.; Cerofolini, L.; Giuntini, S.; Iozzino, L.; Pergola, C.; Sacco, F.; Palmese, A.; Ravera, E.; Luchinat, C.; Baroni, F.; Fragai, M. Epitope Mapping and Binding Assessment by Solid-State NMR Provide a Way for the Development of Biologics under the Quality by Design Paradigm. *J. Am. Chem. Soc.* **2022**, *144* (22), 10006–10016. https://doi.org/10.1021/jacs.2c03232.

(5) Bostock, M.; Nietlispach, D. Compressed Sensing: Reconstruction of Non-Uniformly Sampled Multidimensional NMR Data. *Concepts in Magnetic Resonance Part A* **2017**, *46A* (2), e21438. https://doi.org/10.1002/cmr.a.21438.
(6) Shen, Y.; Delaglio, F.; Cornilescu, G.; Bax, A. TALOS+: A Hybrid Method for Predicting Protein Backbone Torsion Angles from NMR Chemical Shifts. *J. Biomol. NMR* **2009**, *44* (4), 213–223. https://doi.org/10.1007/s10858-009-9333-z.
(7) Pascolutti, R.; Sun, X.; Kao, J.; Maute, R. L.; Ring, A. M.; Bowman, G. R.; Kruse, A. C. Structure and Dynamics of PD-L1 and an Ultra-High-Affinity PD-1 Receptor Mutant. *Structure* **2016**, *24* (10), 1719–1728. https://doi.org/10.1016/j.str.2016.06.026.
(8) Fiser, A.; Šali, A. Modeller: Generation and Refinement of Homology-Based Protein Structure Models. In *Methods in Enzymology*; Charles W. Carter, Jr. and R. M. S., Ed.; Macromolecular Crystallography, Part D; Academic Press, 2003; Vol. Volume 374, pp 461–491.
(9) van Zundert, G. C. P.; Rodrigues, J. P. G. L. M.; Trellet, M.; Schmitz, C.; Kastritis, P. L.; Karaca, E.; Melquiond, A. S. J.; van Dijk, M.; de Vries, S. J.; Bonvin, A. M. J. J. The HADDOCK2.2 Web Server: User-Friendly Integrative Modeling of Biomolecular Complexes. *Journal of Molecular Biology* **2016**, *428* (4), 720–725. https://doi.org/10.1016/j.jmb.2015.09.014.
(10) Honorato, R. V.; Koukos, P. I.; Jiménez-García, B.; Tsaregorodtsev, A.; Verlato, M.; Giachetti, A.; Rosato, A.; Bonvin, A. M. J. J. Structural Biology in the Clouds: The WeNMR-EOSC Ecosystem. *Frontiers in Molecular Biosciences* **2021**, *8*.
(11) Zak, K. M.; Kitel, R.; Przetocka, S.; Golik, P.; Guzik, K.; Musielak, B.; Dömling, A.; Dubin, G.; Holak, T. A. Structure of the Complex of Human Programmed Death 1, PD-1, and Its Ligand PD-L1. *Structure* **2015**, *23* (12), 2341–2348. https://doi.org/10.1016/j.str.2015.09.010.
(12) Xue, L. C.; Rodrigues, J. P.; Kastritis, P. L.; Bonvin, A. M.; Vangone, A. PRODIGY: A Web Server for Predicting the Binding Affinity of Protein-Protein Complexes. *Bioinformatics* **2016**, *32* (23), 3676–3678. https://doi.org/10.1093/bioinformatics/btw514.
(13) Vangone, A.; Bonvin, A. M. Contacts-Based Prediction of Binding Affinity in Protein–Protein Complexes. *eLife 4*. https://doi.org/10.7554/eLife.07454.
(14) Laveglia, V.; Giachetti, A.; Cerofolini, L.; Haubrich, K.; Fragai, M.; Ciulli, A.; Rosato, A. Automated Determination of Nuclear Magnetic Resonance Chemical Shift Perturbations in Ligand Screening Experiments: The PICASSO Web Server. *J. Chem. Inf. Model.* **2021**, *61* (12), 5726–5733. https://doi.org/10.1021/acs.jcim.1c00871.
